# Whole genome sequencing of *Borrelia miyamotoi* isolate Izh-4: reference for a complex bacterial genome

**DOI:** 10.1186/s12864-019-6388-4

**Published:** 2020-01-06

**Authors:** Konstantin V. Kuleshov, Gabriele Margos, Volker Fingerle, Joris Koetsveld, Irina A. Goptar, Mikhail L. Markelov, Nadezhda M. Kolyasnikova, Denis S. Sarksyan, Nina P. Kirdyashkina, German A. Shipulin, Joppe W. Hovius, Alexander E. Platonov

**Affiliations:** 1 Central Research Institute of Epidemiology, Moscow, 111123 Russia; 2 Federal State Budget Scientific Institution “Federal Scientific Center VIEV”, Moscow, Russia; 3 Bavarian Health and Food Safety Authority, German National Reference Centre for Borrelia, Veterinärstr. 2, 85764, Oberschleissheim, Germany; 4 Amsterdam University Medical Centers, location Academic Medical Center, University of Amsterdam, Amsterdam, The Netherlands; 5 Izmerov Research Institute of Occupational Health, Moscow, Russia; 6 Chumakov Federal Scientific Center for Research and Development of Immune-and- Biological Products of Russian Academy of Sciences, Moscow, Russia; 7 Izhevsk State Medical Academy, Izhevsk, Russia; 8 Center of Strategical Planning and Management of Biomedical Health Risks of the Ministry of Health, Moscow, Russia

**Keywords:** Borrelia miyamotoi, Plasmids, Reference genome, Whole genome sequencing, Long-read sequencing

## Abstract

**Background:**

The genus *Borrelia* comprises spirochaetal bacteria maintained in natural transmission cycles by tick vectors and vertebrate reservoir hosts. The main groups are represented by a species complex including the causative agents of Lyme borreliosis and relapsing fever group *Borrelia*. *Borrelia miyamotoi* belongs to the relapsing fever group of spirochetes and forms distinct populations in North America, Asia, and Europe. As all *Borrelia* species *B. miyamotoi* possess an unusual and complex genome consisting of a linear chromosome and a number of linear and circular plasmids. The species is considered an emerging human pathogen and an increasing number of human cases are being described in the Northern hemisphere. The aim of this study was to produce a high quality reference genome that will facilitate future studies into genetic differences between different populations and the genome plasticity of *B. miyamotoi*.

**Results:**

We used multiple available sequencing methods, including Pacific Bioscience single-molecule real-time technology (SMRT) and Oxford Nanopore technology (ONT) supplemented with highly accurate Illumina sequences, to explore the suitability for whole genome assembly of the Russian *B. miyamotoi* isolate, Izh-4. Plasmids were typed according to their potential plasmid partitioning genes (PF32, 49, 50, 57/62). Comparing and combining results of both long-read (SMRT and ONT) and short-read methods (Illumina), we determined that the genome of the isolate Izh-4 consisted of one linear chromosome, 12 linear and two circular plasmids. Whilst the majority of plasmids had corresponding contigs in the Asian *B. miyamotoi* isolate FR64b, there were only four that matched plasmids of the North American isolate CT13–2396, indicating differences between *B. miyamotoi* populations. Several plasmids, e.g. lp41, lp29, lp23, and lp24, were found to carry variable major proteins. Amongst those were variable large proteins (Vlp) subtype Vlp-α, Vlp-γ, Vlp-δ and also Vlp-β. Phylogenetic analysis of common plasmids types showed the uniqueness in Russian/Asian isolates of *B. miyamotoi* compared to other isolates.

**Conclusions:**

We here describe the genome of a Russian *B. miyamotoi* clinical isolate, providing a solid basis for future comparative genomics of *B. miyamotoi* isolates. This will be a great impetus for further basic, molecular and epidemiological research on this emerging tick-borne pathogen.

## Background

*Borrelia miyamotoi* was first discovered in *Ixodes persulcatus* in Japan and described in 1995 [1]. Subsequently it was discovered to be occurring sympatrically with *B. burgdorferi* sensu lato in several *Ixodes* species that also transmit Lyme disease spirochetes. These included *Ixodes persulcatus* in Eurasia [2–7], *I. scapularis* [8–11] and *I. pacificus* [12–15] in North America, and *I. ricinus* in Europe [16–20]. The prevalence of *B. miyamotoi* in ticks was found to be usually lower than that of *B. burgdorferi* s.l. although prevalences of ~ 15% have been reported in some regions [3, 7, 10, 16, 17, 21, 22]. Rodents have been implicated as reservoir hosts for *B. miyamotoi* [23, 24], but transovarial transmission is also known to occur [25, 26] and may contribute to the persistence of this *Borrelia* in nature.

Despite its co-occurrence with *B. burgdorferi* s.l. in hard-bodied *Ixodes* ticks*,* genetic and phylogenetic analyses showed that *B. miyamotoi* belongs to the clade of relapsing fever (RF) spirochetes [1, 2, 16, 23, 27], which are usually transmitted by soft ticks (*Argasidae*) or lice. Similar to other relapsing fever species, *B. miyamotoi* possesses genes encoding variable large proteins and variable small proteins (Vlp and Vsp, respectively) [11, 28, 29]. Vlp and Vsp are expressed during the vertebrate phase of the life cycle of relapsing fever spirochetes. These proteins belong to an antigenic variation system of the spirochetes that permits escape of the hosts’ acquired immune response. This can prolong presence of the spirochetes in the blood stream of an infected animal, thus increasing the opportunity of transmission to a vector [30, 31]. Genetic studies on field-collected samples suggested that there is little genetic variability of *B. miyamotoi* isolates within the population of a single tick species, whilst *B. miyamotoi* isolates from different tick species appeared genetically heterogeneous [3, 22]. Thus, it was suggested that the species *B. miyamotoi* consists of Asian, European, North American - West and East Coast - ecotypes/genotypes [2, 8, 16, 32, 33].

The first cases of human disease caused by *B. miyamotoi* were reported in 2011 in Russia [3]. In that study, 46 cases of *B. miyamotoi* disease (BMD) were described with clinical manifestations that included fever and an influenza-like illness, with myalgia and arthralgia amongst other symptoms. Since then, several hundred BMD cases were identified in Russia [34, 35]. BMD cases have been reported in Europe and the USA as well, but not with such frequency [2, 36–39]. Cases that were reported from Western Europe often involved immunocompromised individuals, but more recently also immunocompetent persons [40, 41]. The widespread geographic distribution of this emerging human pathogen that can utilize many different vectors and hosts, as well as the different clinical presentation of BMD, varying in clinical significance from asymptomatic infection to severe effects such as meningoencephalitis, imply the need to understand the genetic basis of this diversity.

However, compared to other bacterial genomes, *Borrelia* genomes are unusually complex, consisting of a linear chromosome and a number of linear and circular plasmids. Plasmid content and structure does not only vary amongst species, but also may vary within species. Thus the assembly of the complete *B. miyamotoi* genome is a challenging task.

So far, the genome of one *B. miyamotoi* isolate FR64b of the Asian subtype and four American isolates (CT13–2396, CA17–2241, LB2001, CT14D4) have been sequenced [11, 14, 33, 42]. However, a long-read sequencing method was used only for the characterization of CT13–2396. Therefore the number and content of plasmids is not described properly for the other four strains [43].

In the current study, we sequenced the genome of one Russian *B. miyamotoi* patient isolate. The aim of our study was to produce a high quality genome for *B. miyamotoi* in order to provide a reference for further studies into the genetic diversity and the genome plasticity of *B. miyamotoi*. To this end, we evaluated several sequencing and bioinformatics methods, as well as several methods for identification and classifying plasmids. We compared and combined different long-read methods (Pacific Biosciences single-molecule real-time technology (SMRT) and Oxford Nanopore Technology (ONT)) and supplemented assemblies with accurate Illumina short-read sequences. The resulting reference genome will help to simplify and improve future genomic analysis of *B. miyamotoi* isolates, in particular to investigate specific genomic features of Asian *B. miyamotoi* isolates and to identify and investigate virulence and pathogenicity factors.

## Results

### PFGE analysis of *B. miyamotoi* Izh-4 strain

**Fig. 1:**
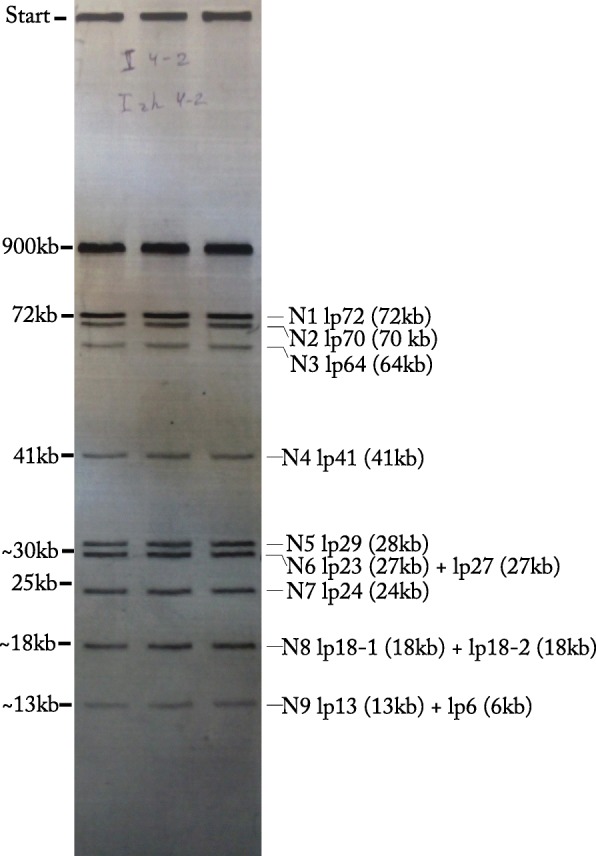
PFGE pattern of chromosomal and plasmid DNA of *B. miyamotoi* isolate Izh-4 in three independent repetitions. N1-N9 indicate PFGE fragments which were subjected to gel extraction and sequencing via the Illumina platform. The name of plasmids with corresponding length is given on the right site of the gel. It was based on the comparison of assembled contigs from each of the PFGE fragments with the final assembly. Of note, the lp6 plasmid did not separate in PFGE, no distinct band at that size was visible. This may have been due to insufficient PFGE conditions, as lp6 sequences were identified in the fragment of 13 kb together with plasmid lp13 by direct sequencing

Pulsed-field Gel Electrophoresis (PFGE) analysis revealed a chromosome with a length of ~ 900 kb and nine non-chromosomal fragments (potential plasmids) (Fig. 1). The first three non-chromosomal fragments with sizes ranging from 72 kb to 64 kb were similar among all Russian *B. miyamotoi* isolates [44] (data not shown). The remaining bands indicated the presence of additional six plasmids with sizes ranging from approx. 40 kb to 13 kb. This is probably an underestimate, since it is well known that plasmids with similar sizes or circular plasmids (which may have different migration patterns than linear plasmids) may not be identified by PFGE.

### *B. miyamotoi* strain, genome sequencing and assembly

In order to obtain a high quality reference genome for comparative genomics of *B. miyamotoi*, the genome of isolate Izh-4 was randomly chosen from available Russian clinical isolates [44] **(**Additional file 1: Table S1**)** and sequenced using different sequencing platforms including Illumina MiSeq and HiSeq, ONT MinION, and Pacific Biosciences SMRT. Assemblies of long reads were corrected using long reads (e.g. PacBio with PacBio; ONT with ONT) and subsequently using highly accurate Illumina sequence reads by means of the Pilon pipeline [45].

Using the MinION platform we obtained 129,992 raw reads of an average length of 6.6 kb. After correction and trimming in the Canu v1.7 pipeline the number of long reads decreases to 31,584 with an average length 7.3 kb. The assembly showed 16 contigs with lengths ranging from 900 kb to 10 kb. Manual validation revealed that two of them - tig00009030 and tig00000013 – were characterized by a specific coverage pattern of ONT reads in two peaks indicating that two separate plasmids were merged. Moreover, the two contigs were 46 kb and 50 kb in size, which was not in line with the PFGE analysis (Additional file 2: Figures S1-S3). Therefore, these contigs were split into two contigs and processed as separate plasmids. In addition, three of the resulting 18 contigs were characterized by low long read coverage (2-3x) and had a high similarity level (≥ 95%) to other contigs and were therefore removed from further analysis. Finally, two of the 15 remaining contigs were automatically circularized with lengths of 30 kb and 29 kb. To summarize, using this method, in the end we obtained 15 contigs corresponding to one main chromosome and 14 potential plasmids, with coverage by trimmed reads ranging from 300x to 20x (Table 1).

**Table 1:** The final composition of *B. miyamotoi* Izh-4 genome and coverage by long and short reads

	GenBank accession numbers	Molecule name	Length, bp	PacBio read coverage before and after correction in brackets	MinION read coverage before and after correction in brackets	Illumina reads coverage
1	CP024390.1	chromosome	906,129	695x (16x)	668x (200x)	440x
2	CP024391.1	pIzh4-lp72	73,492	1378x (24x)	1118x (300x)	402x
3	CP024392.1	pIzh4-lp70	70,072	677x (17x)	573x (122x)	359x
4	CP024401.2	pIzh4-lp64	64,141	321x (5x)	365x (67x)	262x
5	CP024393.1	pIzh4-lp41	41,127	509x (11x)	447x (54x)	523x
6	CP024395.1	pIzh4-cp30–1	30,091	1712x (26x)	591x (162x)	192x
7	CP040828.1	pIzh4-cp30–2	29,490	657x (13x)	265x (49x)	177x
8	CP024396.1	pIzh4-lp29	28,667	1211x (23x)	544x (72x)	614x
9	CP024397.1	pIzh4-lp23	27,717	528x (16x)	329x (37x)	504x
10	CP024398.1	pIzh4-lp27	26,599	862x (17x)	334x (20x)	251x
11	CP024399.2	pIzh4-lp24	24,033	1263x (18x)	470x (78x)	466x
12	CP024400.2	pIzh4-lp18–2	18,334	1554x (17x)	323x (63x)	722x
13	CP024405.2	pIzh4-lp18–1	18,024	771x (14x)	123x (49x)	527x
14	CP024404.1	pIzh4-lp13	13,410	480x (9x)	118x (43x)	327x
15	CP024407.1	pIzh4-lp6	5851	578x (3x)	138x (92x)	625x
			Total reads:	312,224 (2625)	129,992 (31,584)	2,642,950
			Mapped reads:	95% (100%)	100% (100%)	93%

Raw and trimmed\corrected long reads from MinION and PacBio as well as short reads from Illumina were mapped to the final assembly of Izh-4 genome by mininap2 (https://github.com/lh3/minimap2) with default parameters for each type of reads

Using the PacBio platform we obtained 312,224 raw reads with an average length of 4 kb. Using 2635 corrected reads with an average length of 8.8 kb 20 contigs were assembled, with a contig length varying from 6 kb to 906 kb. Three low-coverage contigs, with sequences present in other parts of the genome, were assumed to be assembly artifacts and were removed. Two contigs were manually circularized based on overlapping ends.

Mismatches between ONT and PacBio assemblies were noted and differences to hypothetical lengths of plasmids in PFGE were observed. PacBio unitig#3 was 68 kb in size and was not identified in PFGE. It was similar to three separate ONT contigs (41 kb, 27 kb and 22 kb) (Additional file 2: Figure S4). Three PacBio unitigs corresponding to an ONT contig of 70 kb were identified, so ONT contig was mistakenly split into three separate PacBio contigs (Additional file 2: Figure S5). Moreover, two of these PacBio unitigs #20 (~ 38 kb) and #22 (~ 38 kb) were not observed in PFGE. The 64 kb ONT contig was partially represented in unitig#10, which was 43 kb in size (Additional file 2: Figure S6) and also not found in PFGE. These mis-assemblies of PacBio sequences might have been due to a low amount of DNA submitted for sequencing (1.2 μg), which was lower than requested by the sequencing service (5–10 μg) and did not permit BluePippin size selection. Nonetheless, the remaining contigs were similar between PacBio and ONT assemblies. ONT contigs that were split based on coverage analysis were confirmed by PacBio unitigs as separate sequences. Overall, the extracted consensus sequences from PacBio and ONT assemblies (corrected by using highly accurate Illumina reads) resulted in a complete genome consisting of a chromosome of ~ 900 kb, and 14 putative plasmid contigs, of which two were circular and 12 linear, ranging in length from 6 to 73 kb.

The contigs of the above-described final assembly was also compared with the contigs obtained by direct sequencing of DNA fragments extracted from the agarose gel after separation by PFGE. These contigs were matched using Mummer and visualized by Circos. A number of contigs were produced for the different bands, but only a subset in each band represented the plasmid in question (see Fig. 1 and Additional file 2: Figures S7-S15). For example, for the PFGE fragment N1, 85 contigs were assembled from Illumina short reads, but only one contig of a length of 72,707 bp completely reproduced the lp72 plasmid in the final assembly. Although we were able to identify the majority of linear plasmids by direct sequencing of PFGE fragments, among the collected contigs no sequences corresponding to circular plasmids (cp30–1 and cp30–2) were found. Two of the plasmids, namely lp70 and lp64, were highly fragmented. Many small contig with low k-mer coverage compared to major contigs were observed and were possibly the result of sample contamination during the DNA isolation process.

The final composition of genome is summarized in Table 1. This assembly was deposited in GenBank, BioSample SAMN07572561.

### Determination of telomere sequences on the left and right ends of linear replicons

The genome of isolate Izh-4 of *Borrelia miyamotoi* contains 13 linear replicons. As palindromic sequences were reported at the ends of linear plasmids in other *Borrelia* species [46] we searched whether the linear replicons were flanked with palindromic sequences that resemble short telomere structures forming covalently closed hairpins. When analyzing the terminal regions of the assembled chromosome and linear plasmids, terminal nucleotide sequences were identified, which are presented in Table 2. Identical palindromic sequences were found for lp70R and lp18–1 L, lp70L and lp13L, lp64L and lp41L, lp29R/lp24L/lp23R, lp29L and lp27L, lp24R and lp18–2 L. The lp6L sequence - although palindromic - might not have been identified properly as there was no “signature” sequence. 
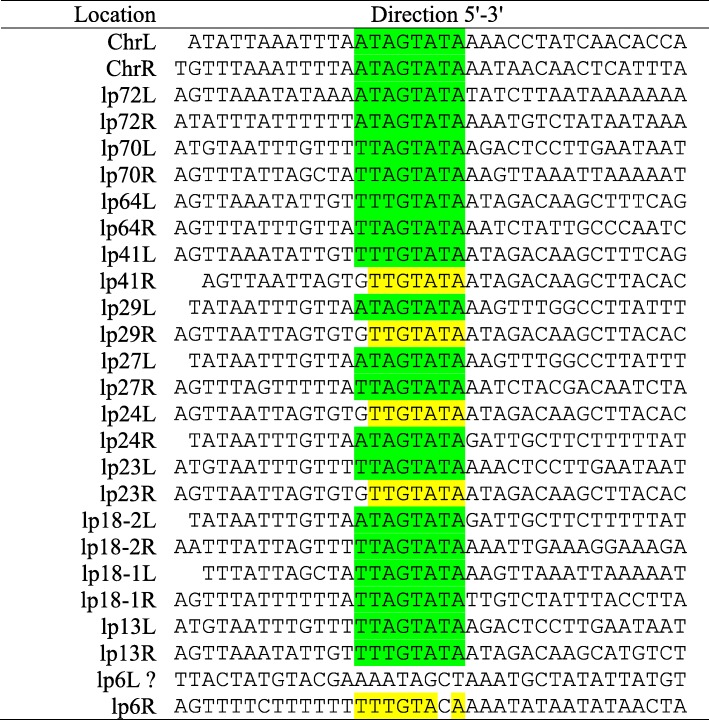

**Table 2:**
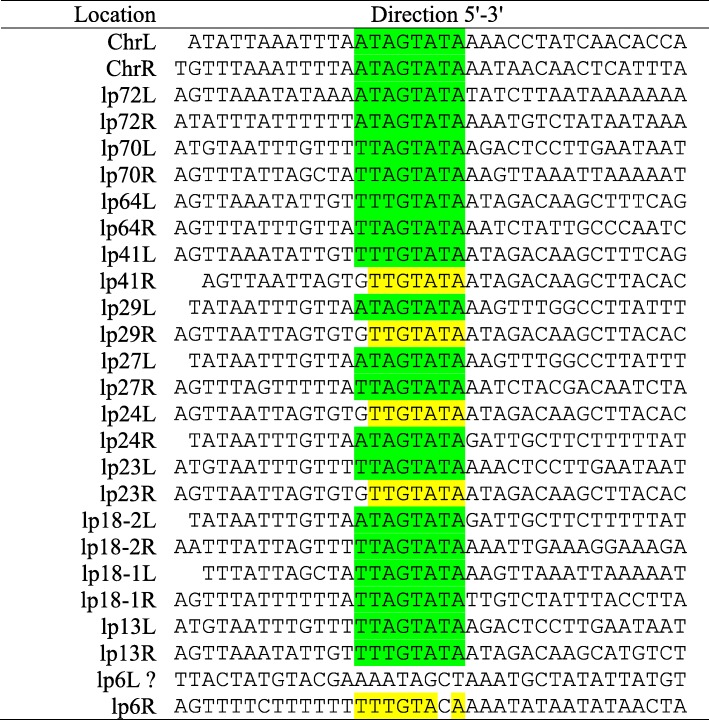
Telomere sequences of chromosome and linear plasmids of isolate *Borrelia miyamotoi* Izh-4

The sequences are oriented such that their hairpin bend would be positioned to their left side. The sequence motif described as “Box 3” is highlighted by green background. The partly identified sequence motif of “Box 3” is highlighted by yellow background. “?” - indicate telomere sequence which might not have been identified properly

Due to the absence of detailed information about telomere sequences for relapsing fever *Borrelia*, and in particular *B. miyamotoi*, we can only suppose that there is evidence for the presence of “Box 3” with the consensus motif “WTWGTATA” starting from position 14, as previously described for Lyme disease *Borrelia* [46–48]. The sequence described as “Box 3” corresponds to a previously annotated conserved region (Box 3), which was assumed to be directly involved in interaction with the telomere resolvase ResT [49, 50].

### Genome content

Genome annotation of isolate Izh-4 revealed a total of 1362 genes including 31 genes for transfer RNA (tRNA), one cluster of three genes of ribosomal RNA (rRNA) (5S, 16S, 23S) and three genes of non-coding RNA (ncRNA). Out of the 1362 genes, 1222 have been annotated as protein-coding genes. The analysis showed the presence of 103 (7.5%) pseudogenes in the Izh-4 genome (Table 3). The majority of pseudogenes were the result of a frameshift. The number of pseudogenes differed between genomic elements and ranged from 0 to 24. The highest number of pseudogenes was present in two plasmids, lp70 and lp64, and in the chromosome, with 24, 23 and 22 pseudogenes, respectively.

**Table 3:** Gene content analysis of Izh-4 genome

	Length, bp	Total genes	Total CDS	COG classified genes	% of COG classified genes	Total number of pseudogenes	% pseudogenes of total genes	Pseudogenes frameshifted	Pseudogenes uncompleted	Pseudogenes with internal stop
chromosome	906,129	850	791	641	81	22	3	17	2	3
lp72	73,492	72	70	15	21	2	3	2	0	0
lp70	70,072	93	69	10	14	24	26	17	5	2
lp64	64,141	84	61	9	14	23	27	19	2	2
lp41	41,127	35	33	12	36	2	6	2	0	0
cp30–1	30,091	42	42	5	11	0	0	0	0	0
cp30–2	29,490	42	41	6	14	1	2	0	1	0
lp29	28,667	23	17	1	5	6	26	4	2	0
lp23	27,717	23	20	2	10	3	13	3	0	0
lp27	26,599	31	27	4	14	4	13	1	3	0
lp24	24,033	20	16	1	6	4	20	4	0	0
lp18–1	18,334	18	10	1	10	8	44	5	2	1
lp18–2	18,024	13	10	1	10	3	23	1	1	1
lp13	13,410	10	9	2	22	1	10	1	0	0
lp6	5851	6	6	1	16	0	0	0	0	0
Total:		1362	1222	711		103				

Functional classification of proteins by comparison with previously defined clusters of orthologous groups (COG) showed that approximately 81% of chromosomal proteins and only 16% of the plasmid proteins of Izh-4 could be assigned to 25 different COG categories (RPS-BLAST, threshold *E*-value 0.01). This confirms that the chromosome is well conserved. Indeed, a comparison based on COG between the chromosomes of Russian isolates with the previously sequenced genomes of the American (CT13–2396) and Asian (FR64b) genotypes did not reveal significant differences either.

The high percentage of COG-classified proteins localized on some plasmids indicates that some plasmids carry vital genes that likely encode proteins that contribute to basic metabolic processes. For example, according to our analysis plasmid lp41 (41 kb) encodes 12 COG-classified proteins, and the three plasmids lp72, lp70 and lp64 encode 15, 10 and 9 of such proteins, respectively (Table 3). It is worth mentioning that lp41 is the main virulence plasmid carrying and expressing the “main variable surface proteins” (variable major proteins, Vmps) [28].

### *Borrelia miyamotoi* chromosome

Pairwise sequence comparison of the linear chromosome of Izh-4 with the previously sequenced genomes of FR64b (Japan), CT14D4, LB2001, and CT13–2396 (USA) of *B. miyamotoi* revealed that the average nucleotide identity (ANI) between chromosomes of Izh-4 and FR64b amounted to 99.97% and to 97.77% to isolates from the USA. Whole genome alignment of these chromosomes did not reveal any noticeable genomic rearrangements such as long insertions\deletions, duplications of regions, and translocations, confirming the conservative nature of the *B. miyamotoi* linear chromosome. However, small differences were detected in polymorphisms of tandem repeats (VNTR), single nucleotide polymorphisms (SNPs), and small indels (Additional file 3: Figures S30 – S31 and Table S2). The total number of differences detected among chromosomes was - unsurprisingly - different between isolates from different geographic regions: Izh-4 and isolates from the USA showed an average of 18,563 differences; Izh-4 and the Japanese isolate had merely 122. The majority of differences were base substitutions. We also identified five sites containing VNTRs (Additional file 3: Figure S30). Such differences may be useful for developing future subtyping schemes for *B. miyamotoi* clinical isolates.

### Plasmid typing by analysis of paralogous gene families (PF) genes

The identified 14 plasmid contigs and the chromosome of Izh-4 were subjected to an analysis to define the type of partition proteins and to decide on potential names for particular plasmids. In order to identify genes homologous to the plasmid replication/maintenance proteins PF 32, 49, 50, 62 and 57 [51, 52], extracted nucleotide sequences of open reading frames (ORFs), including genes annotated as pseudogenes, from the Izh-4 genome as well as reference genomes of different *Borrelia* species were submitted to interproscan annotation and used for comparative phylogenetic analysis (See the Methods section for a more detailed description).

**Fig. 2:**
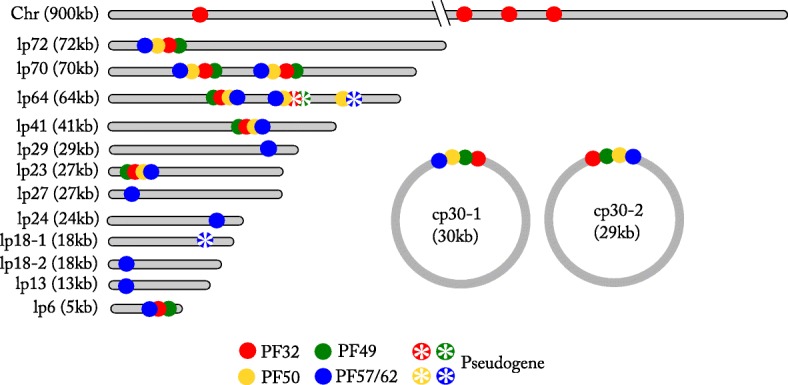
Schematic representation of the Izh-4 segmented genome with identified PF genes 32, 49, 50, 57/62. The order and relative position of these genes on plasmids are displayed

We identified that Izh-4 possessed contigs characterized by different PF genes (Fig. 2). Using a method that was previously described for *B. burgdorferi* [51], we defined the plasmid types in Izh-4 by investigating the phylogenetic relatedness of PF genes to reference genomes. PF genes 32, 49, 50, 57/62 found on the chromosome and several plasmids (lp72, lp41, lp23, lp6) were phylogenetically closely related and formed monophyletic clades to PF genes corresponding to plasmids of genome CT13–2396 **(**Additional file 4: Figures S37 – S40**)**. Despite the fact that in Izh-4 a plasmid of 27 kb length had the same PF genes as the plasmid named lp23 in CT13–2396, we choose the same name for these plasmids which is in accordance to plasmid typing in *B. burgdorferi* sl [51]. Notably, PF genes of Izh-4 and FR64b clustered together in more cases than they did with CT13–2396, indicating a closer genetic/genomic relatedness of Russian and Japanese *B. miyamotoi* isolates than of Russian and North American isolates (including plasmid content).

We found two plasmids - lp70 and lp64 - that have not previously been described in *Borrelia*. Each of these plasmids carried several sets of PF genes suggesting that they were formed by fusion of different types of plasmids in the past. Plasmid lp70 of Izh-4 carried two copies of PF32, which phylogenetically clustered with plasmid contigs of FR64b. However, one of the copies showed high similarity to the PF32 of plasmid cp2 of CT13–2396 **(**Additional file 4: Figure S37**)**. Plasmid lp64 carried three sets of PF 32, 49, 50, 57/62. Of these one cluster was represented only by PF50 while PF57/62 was a pseudogene and PF32 and PF49 were absent. The other two sets of genes had four PF genes, but one set was characterized by the presence of pseudogenes related to PF 32 and 49 (Fig. 2). Two copies of PF32 of lp64 clustered in different phylogenetic groups and similar copies were found in the FR64b genome. One of the copies of lp64-PF32 is most similar to PF32 located on plasmid pl42 of *B. duttonii* isolate Ly; the other copy (pseudogene) is most similar to PF32 located on plasmids lpF27 of *B. hermsii* HS1 and lp28–7 of *B. afzelii* PKo **(**Additional file 4: Figure S37**)**.

Plasmids lp29, lp27, lp24, lp18–2, and lp13 possessed only one copy of PF57/62, but the copy in plasmid lp18–1 was a pseudogene of PF57/62. This was consistent with data from previously sequenced genomes [11]. For instance, *B. miyamotoi* CT13–2396 plasmids lp30, lp20–1, lp20–2 and lp19 have only the PF57/62 gene, and plasmid cp4 only carried a PF50 **(**Additional file 4: Figure S39, S40**)**. Although the classification of plasmid compatibility types was mainly based on the phylogeny of the PF32 locus, in cases where this locus was absent, we used PF57/62 for plasmid typing. In the phylogeny of PF57/62, plasmids lp29, lp27, lp24, lp18–2, and lp13 of Izh-4 and other *B. miyamotoi* isolates formed a clade distinct from most other RF and LB species, except for *B. hermsii* HS1 lpG27. Near identical PF57/62 were found for two pairs of plasmids of Izh-4: plasmids lp29 - lp27 and lp18–1 - lp18–2. This could raise the question whether these are indeed different plasmids. However, these pairs of plasmids had no other extended regions of nucleotide similarity **(**Additional file 3: Figures S33, S34) beyond the PF57/62 locus, indicating they are two different pairs of plasmids. PF57/62 of plasmid lp13 clustered together with the PF57/62 of lp30 of CT13–2396 and a gene located on a plasmid contig (CP004259.1) of FR64b. The PF57/62 of Izh-4 lp24 was nearly identical to a homologous gene located on a plasmid contigs (CP004252) of FR64b. It should be noted that clustering of plasmids based on PF32 genes correlates with groups of plasmids based on PF57/62 clustering, indicating a similar evolutionary patterns between PF32 and PF57/62. Since we did not identify variants of the PF57/62 genes of previously sequenced *B. miyamotoi* genomes that would be close enough to the PF57/62 genes of the Izh-4 genome, we decided to establish the names of plasmids based on their length.

The analysis allowed us to identify only two circular plasmids, each of which was approximately 30 kb in length. The percentage of identity between them was 79%. The set and relative position of ORFs between these plasmids was collinear, with the exception of the variation in the number of Mlp genes (cp30–1 had two genes, cp30–2 had one gene) and inversion of the gene cluster of PF 32, 49, 50, 57/62. Both plasmids are characterized by the presence of genes encoding PBSX phage terminase large subunit, site-specific integrase, indicating a relationship to prophage-related plasmids [53–55]. In addition, both circular plasmids are characterized by the presence of a complete set of PF 32, 49, 50, 57/62 genes. According to the phylogeny of the PF32 genes, these two plasmids belong to different phylogenetic clusters. The PF32 gene of plasmid cp30–1 was more closely related to the PF32 gene localized on plasmids pl28 (*B. duttonii* Ly) and lp28–8 (*B. afzelii* PKo). In turn, the PF32 gene of plasmid cp30–2 was phylogenetically closest related to the PF32 gene localized on plasmid lpT28 of *B. hermsii* HS1.

### Organization of the lp41 virulence plasmid

**Fig. 3:**
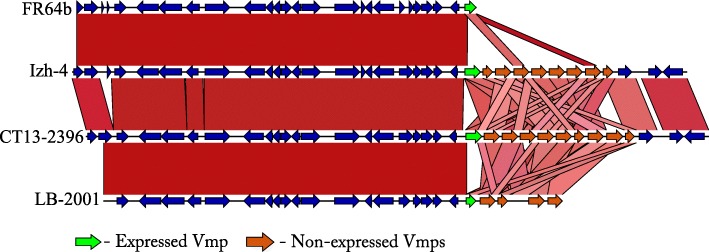
Comparison of the nucleotide sequences of the virulence plasmid lp41 of *B. miyamotoi* isolates originating from North America (LB-2001, СT13–2396), Japan (FR64b), and Russia (Izh-4). Blocks that are colored in red (range of percent identity 100–70%) indicate similar areas between plasmids. The arrows indicate the genes and direction of ORF. The Vmp block of genes is represented by the genes immediately after the expression site - expressed Vmp (light green arrow) and non-expressed Vmp genes (orange arrows). Other ORFs are shown as blue arrows

**Fig. 4:**
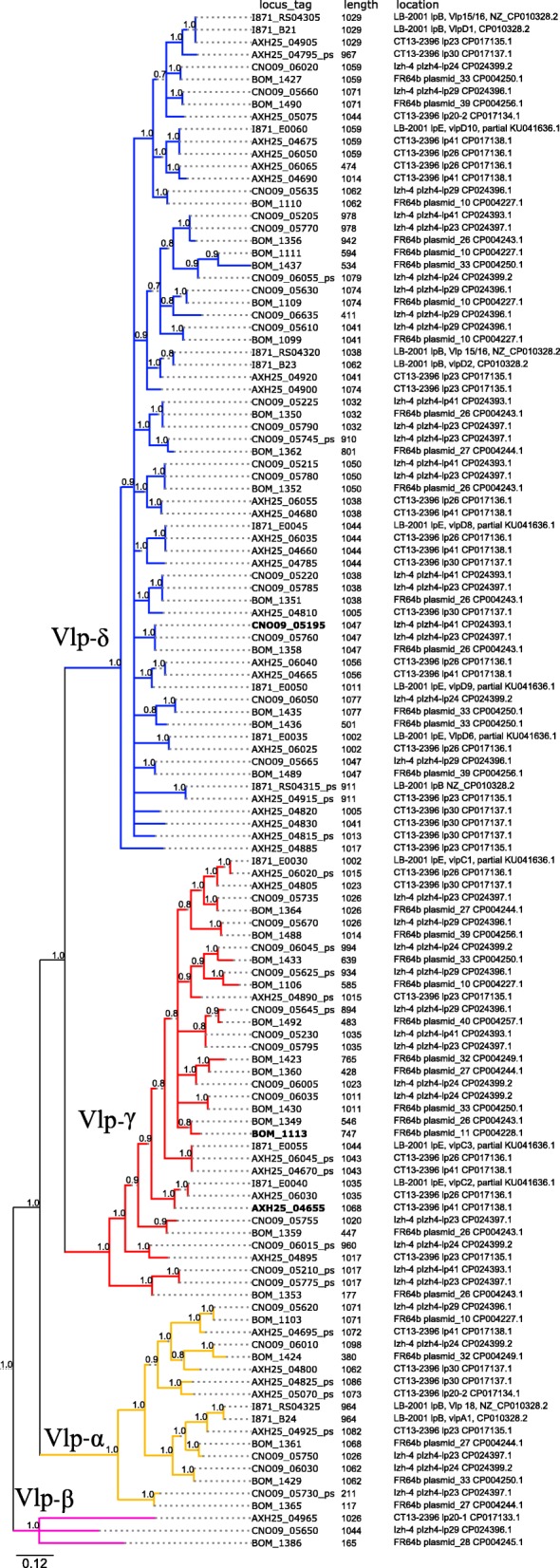
Phylogenetic diversity of Vlp genes in Izh-4, FR64b, CT13–2396 and LB-2001 genomes. Different colors of nodes indicate different Vlp-subfamilies: yellow - Vlp-α, red - Vlp-γ, blue - Vlp-δ, purple - Vlp-β. ORFs marked by bold font are genes located after the expression site. Locus tags of pseudogenes are marked by the postfix “_ps”. The tree was constructed based on pairwise alignment of nucleotide sequences of ORFs/pseudogenes which contain domains corresponding to the lipoprotein_2 family (PF00921) or the Variable surface antigen VlsE superfamily (SSF74748). Vlp-β genes were used as outgroup to root the tree

**Fig. 5:**
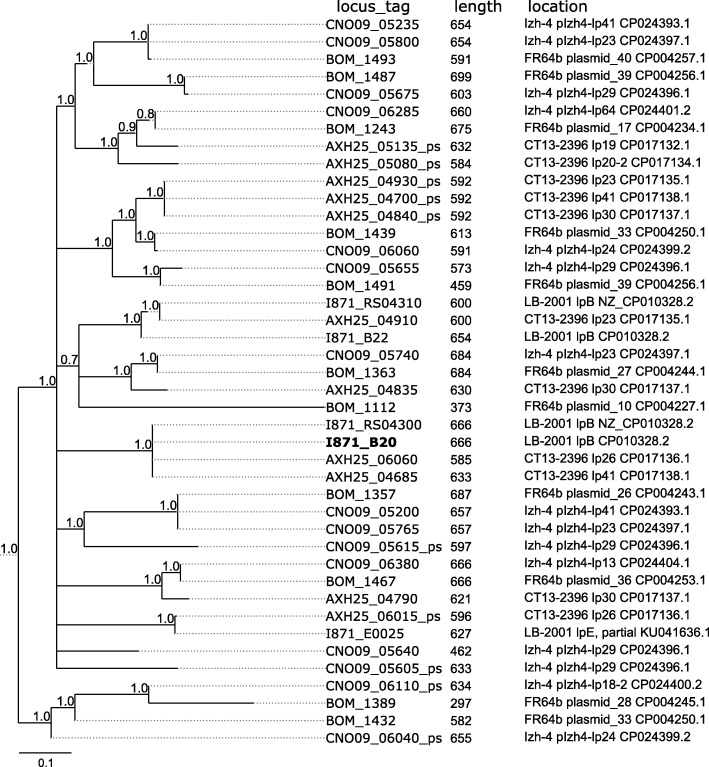
Phylogenetic diversity of Vsp genes in Izh-4, FR64b, CT13–2396 and LB-2001 genomes. ORFs marked by bold font are genes located after the expression site. Locus tags of pseudogenes are marked with the postfix “_ps”. The tree was constructed based on pairwise alignments of nucleotide sequences of ORFs/pseudogenes which contain domains corresponding to the lipoprotein_6 family (PF01441) or the outer surface protein C (OspC) superfamily (SSF63515). The tree was midpoint rooted

Plasmid lp41 appears to play a pivotal role in virulence of *B. miyamotoi* by expressing the Vmps, which enable the bacteria to escape the host immune system during infection [28]. We performed a comparison of lp41 plasmids using BLASTn analysis between Izh-4 and earlier sequenced isolates of *B. miyamotoi* from USA (LB-2001 and CT13–2396) and Asia (FR64b). This analysis revealed a high degree of similarity in the relatively conserved 3 ‘and 5’ regions flanking the variable region of the Vmp genes (Fig. 3). Izh-4 carries a gene encoding the Vlp-δ protein (locus tag: CNO09_05195) after the expression site, while genomes FR64b and CT13–2396 carry Vlp-γ (BOM_1113, AXH25_04655) (Fig. 4) and LB-2001 carry Vsp1 (I871_B20) (Fig. 5).

**Fig. 6:**
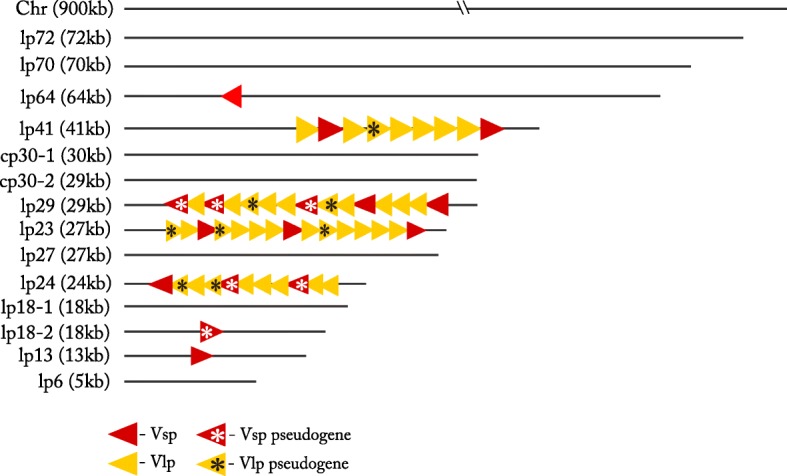
Number and location of Vmp genes in the Izh-4 genome

Some minor 800 bp insertions were detected at the left-end of lp41plasmids between pairs of isolates: FR64b - Izh-4 and CT13–2396 - LB-2001 (data not shown). At the same time, the number and order of the Vmp genes were unique for each of the isolates (partially shown in Fig. 3 and Fig. 6). In addition, single nucleotide variations as well as a 138 bp deletion in an intergenic region before the expression site were detected in both Asian genomes, Izh-4 and FR64b, in comparison to CT13–2396 and LB-2001 (Additional file 3: Figure S35). This might be a marker for differentiation of lp41 plasmids of Asian and American genotypes. Importantly, the organization of the sequence expression site did not differ between *B. miyamotoi* isolates, the nucleotide composition of the Ribosome Binding Site (RBS), the “-10”, and “-35” sites were 100% identical (Additional file 3: Figure S35, bottom), which could be very helpful in identifying the expressed Vmp [28].

### Intragenetic diversity of variable large proteins and variable small proteins

All Izh-4 nucleotide sequences of genes and pseudogenes were searched to assess whether they belonged to the family of lipoproteins in the InterPro database. In total, we found 39 genes encoding variable large proteins (Vlp), nine of them were pseudogenes, and 15 genes encoding variable small proteins (Vsp), including five pseudogenes. Vlp and Vsp genes were clustered in an island manner and were mostly located on plasmids lp41, lp29, lp23 and lp24. Some single Vsp genes were located on lp64, lp18–2 and lp13 plasmids (Fig. 6).

Phylogenetic analysis of the extracted Vlp genes and pseudogenes of four *B. miyamotoi* genomes showed that Vlp genes of Izh-4 formed well supported clades: four clades of Vlp-δ (20 genes), Vlp-γ (13 genes), Vlp-α (five genes) families and one gene on lp29 plasmid corresponded to Vlp-β (Fig. 4). The closest homologues to Vlp-β at 78% amino acid identity were identified in the genomes of *B. crocidurae* DOU (AHH07120.1) and *B. hermsii* (WP_064536660.1). Notably, Vlp-β genes were not described in the genomes of *B. miyamotoi* LB2001 [28], however, similar genes were present in the genome of CT13–2396 (AXH25_04965) and the partially sequenced genome of FR64b genome (BOM_1386) (Fig. 6, lower purple branch).

Phylogenetic analysis of the extracted Vsp genes did not show any patterns of clustering (Fig. 5). However, comparing 14 of Vlp and 4 Vsp genes showed that they are present in two identical copies located on plasmids lp41 and lp23. A BLAST analysis of nucleotide sequences of these plasmids showed that the right parts of plasmids lp41 and lp23 were identical, with the same order of Vlp and Vsp genes and its pseudogenes (Additional file 3: Figure S36). Pairwise comparison of plasmids containing clusters of these genes did not reveal any similarities like the one found between lp41 and lp23. Such right-end similarity of lp41 and lp23 was also detected in CT13–2396.

### Comparison of plasmid sequences among *B. miyamotoi* isolates

To explore the plasmid similarity between different *B. miyamotoi* isolates, we compared the nucleotide sequences of the three isolates CT13–2396, FR64b and Izh-4 (Additional file 2: Figure S15 – S29). We chose these isolates since for CT13–2396 an almost complete genome and for Izh-4 a completed genomes were available and for FR64b a draft genome with 50 contigs was accessible in GenBank. Within these three genomes, we found four common plasmids with high nucleotide similarity: lp72, lp41, lp23 and lp6 (Table 4). Plasmids lp70, lp64, lp27, and lp13 of Izh-4 were only present in the Asian FR64b genome, but absent in the North American isolate CT13–2396. Plasmids cp30–1, cp30–2, lp29, lp24, lp18–1 and lp18–2 were partly present in the F64b genome, and absent in CT13–2396.

**Table 4:** Plasmid comparisons of *B. miyamotoi* strains

Izh-4 plasmids as reference sequences	Matched plasmids of CT13–2396 genome to Izh-4 plasmids	Matched contigs of FR64b genome to Izh-4 plasmids
pIzh4-lp72	lp72	CP004218
pIzh4-lp70	absent	CP004223, CP004233, CP004231, CP004221
pIzh4-lp64	absent	CP004238, CP004234, CP004222, CP004240, CP004239
pIzh4-lp41	lp41	CP004228
pIzh4-cp30–1	absent	partially covered by CP004247 and CP004219
pIzh4-cp30–2	absent	partially covered by CP004220, CP004230 and CP004229
pIzh4-lp29	absent	partially covered by CP004227 and CP004256
pIzh4-lp23	lp23	CP004244, CP004243
pIzh4-lp27	absent	CP004235
pIzh4-lp24	absent	partially covered by CP004250
pIzh4-lp-18-2	absent	partially covered by CP004245
pIzh4-lp18–1	absent	partially covered by CP004251
pIzh4-lp13	absent	CP004253
pIzh4-lp6	lp6	CP004258

### Phylogenetic analyses

#### Phylogeny of Borrelia spp. based on chromosomal genes

**Fig. 7:**
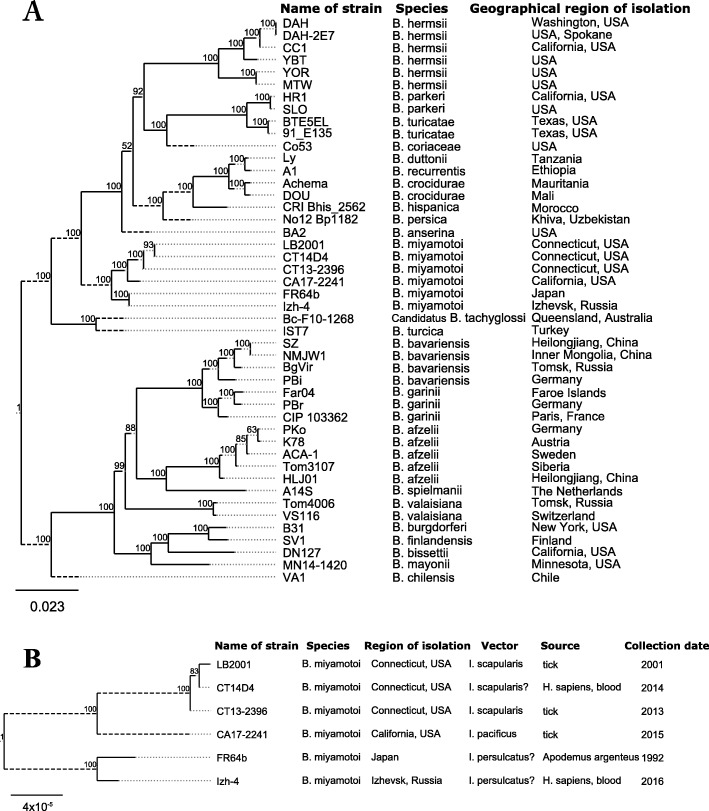
**a** Phylogenetic tree of *Borrelia* species based on the concatenated alignment of nucleotide sequences of 249 core genes located on the chromosome. *Borrelia miyamotoi* clusters with relapsing fever species. **b** Phylogenetic tree of *B miyamotoi* strains based on concatenated alignment of nucleotide sequences of 719 core genes. A maximum likelihood tree was constructed using RAxML software using a nucleotide substitution model with a gamma distribution of variable positions (GTR + Γ). The resulting tree was midpoint rooted using Figtree (http://tree.bio.ed.ac.uk/software/figtree/). Long branches shown not according to scale are indicated by dashed lines. Scale bar indicates substitution rates

To understand the relationships of isolate Izh-4, North American and Asian *B. miyamotoi* isolates as well as to other *Borrelia* species, we performed a phylogenetic analysis of the newly sequenced genome (Izh-4) and *Borrelia* genomes deposited in GenBank (Additional file 1: Table S1). To date, these genomes comprised completed chromosomes and/or several completed plasmids (lp73, lp41, lp23 and lp6). The phylogenetic tree was reconstructed using a concatenated alignment of nucleotide sequences of 249 core genes localized on the chromosome (minimum percent identity for BLASTp 70%) and identified during the process of protein clustering among all *Borrelia* genomes. This phylogenetic analysis showed that *B. miyamotoi* forms a monophyletic clade inside the relapsing fever group and was split into two lineages belonging to the Asian and American genotype. The Asian lineage includes the Izh-4 and FR64b from Japan (Fig. 7a).

For a more detailed analysis, i.e. to determine intraspecific differences among *B. miyamotoi* isolates, we conducted a reciprocal BLASTp search for core genes, but now only within the species *B. miyamotoi*. As a result, 719 orthologous genes were identified (minimum percentage identity for BLASTp 80%) (Fig. 7b).

Mean SNP-distances (in concatenated alignment of core genes) between isolates from Northeast America (CT13–2396, CT14D4, LB-2001), Japan (FR64b) and Russia (Izh-4) were as follows: Northeast American - Russian – 13,767 SNPs, Northeast American – Japanese – 13,776 SNPs, and Russian – Japanese - 36 SNPs. Among the three Northeast American isolates six SNPs were found.

## Discussion

Genetically *B. miyamotoi* has been divided into distinct populations, with population boundaries apparently determined by vector association [2, 8, 16, 32, 33]. To investigate genetic differences in *B. miyamotoi* populations, comparative genomics should be employed, but this requires high quality reference genomes [56]. Although several *B. miyamotoi* draft genomes from North America, Russia and Japan exist [11, 14, 42, 44], a completed genome has not been finished for the Asian genotype of *B. miyamotoi*. Therefore, to provide a basis for comparative genomics studies, here we assembled a reference genome for the Russian *B. miyamotoi* isolate Izh-4 using currently available long-read and short-read technologies.

For some bacteria the assembly of completed genomes had been reported using Nanopore sequencing as sole method [57] or combining long-read with Illumina short-read technology [43, 58, 59]. Initial assemblies combining Illumina and Nanopore reads of *B. miyamotoi* genomes from Russia gave unsatisfactory results, as not all genomic elements (plasmids) were properly assembled [44]. Similar issues were encountered before with *B. miyamotoi* genomes, i.e. some plasmids were incompletely assembled, even though long- (PacBio SMRT) and short-read (Illumina) methods were combined [11]. We therefore sequenced one of the available Russian isolates, Izh-4, using PacBio SMRT technology (in addition to ONT) and compared contigs obtained using the two long-read technologies with results obtained by PFGE. In addition, for accuracy, consensus sequences were generated using Illumina sequence reads. This strategy resulted in a completed reference genome for isolate Izh-4 consisting of one linear chromosome of 906 kb, 12 linear plasmids ranging in size from 6 to 72 kb and two circular plasmids of about 30 kb.

Annotation of the genome of *B. miyamotoi* isolate Izh-4 revealed a total of 1362 ORFs with 37 encoding RNA. The linear chromosome was predicted to encode 850 ORFs and the high level of conservation of the linear chromosome was evidenced by the majority of these loci (81%) being functionally classified as COG. Alignment and comparison of the chromosome with other Russian isolates (data not shown) and isolates from Japan (FR64b) and North America (LB-2001, CT13–2396, CT14D4) confirmed the conservation of the main chromosome. There were neither major nor minor re-arrangements nor insertions/deletions observed in this analysis. As expected, similarity of chromosomes was more pronounced among the Russian and Japanese isolates with fewer SNPs and higher ANI values (ANI 99.9% compared to 97.7% between North American and Russian isolates). SNP values between Russian/Asian and North American isolates were 1000 or 300 fold higher, respectively, than between Asian and Russian isolate.

There was considerably more variation in the plasmids fraction, especially between isolates from different continents. For isolate Izh-4, 12 linear and two circular plasmids were identified. Compared to the chromosome, the percentage of functionally COG classified genes ranged from 22 to 5%, suggesting many more CDS with unique or unknown function in the plasmid fraction. Plasmids with the highest percentages of pseudogenes included lp18–1 (44%), lp64 (27%), lp70 (26%), lp18–2 (23%), and lp24 (20%). In PFGE plasmids with different sizes were observed for the additional five Russian isolates (i.e. Izh-5, Izh-14, Izh-16, Yekat-1 and Yekat-6, data not shown), suggesting that the high conservation observed for the *B. miyamotoi* chromosome does not extend to the plasmid fraction. To better understand the evolutionary relationship of plasmids, we employed a previously described method that focused on plasmid replication/maintenance proteins [51, 52].

Since plasmids in *Borrelia* can vary considerably in size and may undergo intra-specific rearrangements, problems of identifying plasmids due to the similarity of the genomic content exist [51, 60]. In addition, the transition of plasmids from circular to linear or vice versa makes identifying and classifying plasmids within and between *Borrelia* species challenging. The importance of plasmid typing is underpinned by the fact that many genes involved in virulence and pathogenicity of *Borrelia* are plasmid-borne.

Plasmids in the Lyme borreliosis group of spirochetes have been typed and classified using a family of paralogous proteins described as plasmid replication/maintenance proteins [52, 60, 61]. Due to PF32 being homologous to the ParA protein in other bacteria, this group of proteins - termed PF32, PF49, PF50, and PF57/62 – were used to determine the plasmid compatibility type, although in *Borrelia* PF32 may not fulfil this role [52, 61]. In the different plasmids, one or several sets of these protein coding genes were found while in others only subsets of these molecules were identified. This is consistent with data from previous studies which showed that for Lyme borreliosis spirochetes or *B. miyamotoi* isolate CT13–2396 some plasmids carried only the PF57/62 gene [11, 51, 52]. Phylogenetic analyses of these paralogous gene family allowed us to identify plasmids of the same compatibility type (i.e. plasmids with PF genes from the same clade) in draft genomes of North American and Asian *B. miyamotoi* isolates. Perhaps not surprising, given the greater genetic similarity on the chromosome and the genetic homogeneity of *B. miyamotoi* populations in the same vector species [22, 32], more contigs with the same compatibility types of plasmids were found in the Japanese isolate FR64b than in the Connecticut isolate CT13–2396. In the latter isolate four plasmids representing compatibility types to Izh-4 were identified: lp72, lp41, lp23, and lp6. Interestingly, two of these plasmids, lp41 and lp23, contained Vlp and Vsp genes in both isolates. In total in CT13–2396, 23 ORF encoding Vlp proteins were identified in five plasmids whilst in Izh-4 four plasmids were found encoding a total of 38 Vlp (including 9 pseudogenes).

The characterization of other Russian, North American and European *B. miyamotoi* isolates - using the technological and bioinformatic platform shown in the current study - is underway. This will confirm the effectiveness of methodology and help to clarify the evolutionary history of *Borrelia* and to link the genetic peculiarities of *B. miyamotoi* with epidemiological, microbiological, immunological and clinical features of this emerging infection.

## Conclusion

We have assembled a high quality reference genome for a Russian isolate of *B. miyamotoi* and this required employing reads from two long-read and a short-read sequencing technologies. This provides a basis for further investigations to identify similarities / differences between *B. miyamotoi* isolates from different geographical populations and how these may be related to differences in virulence and human pathogenicity.

## Methods

### *Borrelia miyamotoi* isolates and their cultures

Strain Izh-4 was isolated from blood of Russian patient with acute BMD in Izhevsk City in 2016 and cultured in MKP-F medium [62]. DNA was extracted (see the sections below for the different methods used) from about 10^9^ spirochetes after the third in vitro passage.

### Plasmid DNA separation by pulsed-field gel electrophoresis (PFGE)

A standard operating procedure for PulseNet PFGE for Enterobacteria [63] was used with modifications (Additional file 1: Method S1). Nine extrachromosomal fragments ranging from 13 to 73 kb were cut out of the gel and dissolved in Agarose Dissolving Buffer (Zymoresearch), DNA was extracted and subjected to Illumina sequencing.

### Whole genome sequencing and data analysis

#### Illumina sequencing

Total DNA was extracted from borrelia suspension using the DNeasy Blood & Tissue Kit for sequencing using a MiSeq platform. A NexteraXT DNA Library Kit (Illumina, USA) was used for library preparation. DNA-libraries were sequenced using a 500-cycle V2 reagent kit on a MiSeq (Illumina, USA). Low quality reads and adapter sequences were removed from the Illumina reads by BBTools [64]. Assembly was performed by SPAdes-3.9.0 [65].

#### MinION sequencing and data analysis

MinION sequencing (Oxford Nanopore Technologies, UK) was performed by ZF-Genomics, Leiden, The Netherlands. Total DNA of isolate Izh-4 was extracted using the Qiagen Tip-100 prep (Qiagen, Germany). The Native Barcoding Kit 1D (EXP-NBD103) was used together with the Ligation Sequencing Kit (SQK-LSK108) to prepare a Nanopore sequencing library from total DNA. A R9.4 MinION flow cell was used for sequencing. Base calling of MinION sequences was performed using Albacore v1.1.0; adapters were removed by Porechop [66]. Canu v1.7 was used for correction, trimming and de novo assembly of ONT long reads with default parameters and a genome size of 1.6 Mb. Following assembly each contig was inspected for the presence of long inverted repeats at the ends or end to end overlaps using dot plot analysis implemented in FlexiDot [67]. The revealed overlaps were manually verified by alignment using Mafft v7.271 [68] with subsequent removal. Automatic circularization was performed by an APC (A Perfect Circle) script [69] with manual verification. The draft genome assembly was polished by two steps of correction. First, we mapped corrected ONT reads to contigs with Nanopolish [70]. Then we corrected the obtained consensus sequence by mapping Illumina pair-end reads using Pilon v1.22 [45]. Following read mapping the consensus sequences were extracted for further analysis.

#### PacBio sequencing and data analysis

DNA of isolate Izh-4 was submitted to WGS using SMRT sequencing on the Pacific BioScience Technology platform. The sequencing service was provided by the core facility located at the Norwegian Sequencing Centre (NSC) (www.sequencing.uio.no). DNA was extracted from 64 × 10^9^ cells using a Maxwell® 16 and a Maxwell LEV Blood DNA kit (Promega, Germany). The 20 kb library preparation protocol was employed. Size selection of the final library was performed using 0.4x Amp beads. The library was sequenced on a Pacific Biosciences RS II instrument using P6-C4 chemistry with 360 min movie time, two SMRT cells were used for sequencing due to poor loading. De novo assembly was performed using hierarchical genome assembly process (HGAP v3, Pacific Biosciences, SMRT Analysis Software v2.3.0) with default parameters (expected genome size 1.6 Mb, minimum target coverage 15X). RS_Resequencing.1 software (SMRT Analysis version v2.3.0) was used to map SMRT reads back to sequences in order to correct contigs after assembly clean-up. PacBio contigs were polished by mapping Illumina pair-end reads using Pilon v1.22.

#### Determination of the terminal sequences (telomere sequences) of linear replicons

Determination of the terminal sequences (telomere sequences) of the chromosome and linear plasmids was carried out. For identification of telomere sequences (which are represented as palindrome sequences at the right and left ends of linear contigs) de novo assembled PacBio and ONT contigs were used. Each of the linear elements was aligned against itself and dot plot analysis was performed. For each alignment, we determined the coordinates of the breakpoints determined using dot plot analysis, see (Additional file 5: Figure S41). We cut the nucleotide sequence 200–1000 bp above and below the breakpoint and checked for the presence of palindrome sequences using the Einverted tool of the Emboss package [71]. If palindromes were not detected in a contig by dot plot analysis we mapped the trimmed and preassembled PacBio reads onto this contig and used the part of reads spanning the edge of the left or right ends of the contig and analyzed them in Einverted tool.

### Bioinformatics analysis

#### Genome sequences used for comparative analysis

A Table with all isolates used in this study for plasmid typing, comparative genomics and phylogeny is shown in Supplementary information (Additional file 1: Table S1).

#### Calculation of nucleotide identity between *B. miyamotoi* chromosomes

The average nucleotide identity (ANI) between *B. miyamotoi* chromosomes was calculated using the Pyani tool [72] with a BLAST method to align 1020 nt fragments of the input sequences.

#### Determination and visualization of similarity between genomes and plasmids

We used Circos v0.69–5 [73] and Mummer v3.0 [74] to show similarities between different plasmids, contigs or different assemblies. For comparison contigs were aligned all versus all and the results of the alignment were visualized using the Circos tool. Only matched regions with more than 90% identity were taken into account.

To identify differences in the nucleotide sequences of *B. miyamotoi* chromosomes of various isolates NucDiff [75] was utilized.

To determine whether differences may exist in the virulence plasmid lp41 of different isolates, annotated sequences of lp41 plasmids of *B. miyamotoi* isolates were visualized and compared in Easyfig software [76].

#### Genome annotation and designation of plasmid types

Annotation of assembled contigs was performed using a local version of the NCBI Prokaryotic Genome Annotation Pipeline [77]. To identify the plasmid type of particular contigs we used a principle suggested by Casjens et al. [51] that is based on comparison of paralogous gene families (PF) 32, 49, 50, 62 and 57. The name (nomenclature) that we give to particular contigs (plasmids) is based on the relationship of PF genes identified in those contigs with analogous genes/proteins of previously designated plasmids in a set of reference genomes of different *Borrelia* species with high-quality annotation. We inferred the relationship of plasmids found in our study by comparison to previous naming schemes of PF [51] and phylogeny. We searched databases of protein sequences by using InterProScan software to find specific PF proteins in annotated genomes such as the Conserved Domains Database (CDD) [78], Protein Families database (Pfam) [79], database of structural and functional annotation for all proteins and genomes (SUPERFAMILY) [80]. For example, PF32 was found to be homologous to proteins in the CDD database with accession numbers cd02038 and cd02042, PF49 is related to PF01672 in Protein Families database, PF50 relates to PF02890, and PF57/62 to PF02414.

Our comparative analysis included the following steps. (1) We extracted all ORFs’ nucleotide sequences, including ORFs which were identified as pseudogenes using the NCBI Prokaryotic Genome Annotation Pipeline, from our reference and other sequenced genomes and placed them into one file. Reference genomes included *B. burgdorferi* B31 (GCA_000008685.2), *B. afzelii* PKo (GCA_000222835.1), *B. duttonii* Ly (GCA_000019685.1), *B. hermsii* HS1 (GCA_001660005.1), *B. miyamotoi* CT13–2396 (GCA_001767415.1), *B. miyamotoi* FR64b (GCA_000568695.2), and the partially sequenced genome of *Borrelia miyamotoi* LB-2001 (GCA_000445425.4). (2) We clustered sequences using CD-HIT on a 90% level. (3) Each cluster’s representative sequence was subjected to InterProScan analysis to determine whether it matches to a particular family of proteins in CDD, Pfam, or SUPERFAMILY database. (4) Subsequently, we extracted all sequences from the CD-HIT clusters which had their representative matched to specific IDs of specific PF. (5) Afterwards, we performed pairwise sequence alignment and distance tree reconstruction using a pairdist script [81] with 1000 bootstrap replicates which allowed us to understand the relatedness among specific PF genes from reference genomes with known plasmids names and the newly sequenced genome elements in our study. After designation of plasmid types the assembly of chromosome and plasmids were submitted to GenBank.

#### Functional classification of proteins by comparison with previously defined COG

Classification of proteins of the sequenced Izh-4 genome to clusters of orthologous groups (COG) was performed using a Perl script (cdd2cog.pl) from a collection of bac-genomics-scripts [82].

#### Identification and phylogenetic analysis of Vmp genes

As an independent and additional in silico analysis, we extracted all nucleotide sequences of ORFs (CDS and pseudogenes) from *B. miyamotoi* isolate Izh-4. We next subjected all these sequences to InterProScan analysis using the InterProScan match lookup service version 5.23–62.0, with a search against Pfam and SUPERFAMILY databases as an option. We subsequently retrieved all matches to PF01441, SSF63515 **(**Vsp proteins) or PF00921, SSF74748 **(**Vlp proteins**)** families. Finally, pairwise alignments of nucleotide sequences and phylogenetic analyses were performed using the pairdist script with 1000 bootstrap repetitions. The phylogenetic tree was visualized using Ete3 Python module.

#### Phylogenies

Identification of orthologous gene cluster and the production of a core genome alignment of chromosomes or particular plasmids was carried out using Roary v1.007002 [83]. For interspecies comparison among *Borrelia* chromosomes, a minimum of 70% identity for BLASTp searches was used, for intraspecies comparison of *B. miyamotoi* chromosomes this value was set to 95%. A phylogenetic tree was inferred based on core genome alignments using RAxML v8.2.9 with GTR + Γ nucleotide substitution model and 1000 bootstrap replicates. The phylogenetic tree was visualized using Python v2.7.11 and the Ete3 Python module.

## Supplementary information

**Additional file 1: Table S1.** Strains used in this study with NCBI Biosample accession numbers.

**Additional file 2: Method S1.** Plasmid DNA separation by Pulsed-field Gel Electrophoresis (PFGE). **Figures S1 - S6.** Visual comparisons of ONT, PacBio and final assemblies of plasmids of *Borrelia miyamotoi* isolate Izh-4. **Figures S7 - S15.** Visual comparisons of contigs assembled from short Illumina reads for each PFGE fragment and final assemblies of plasmids of *Borrelia miyamotoi* isolate Izh-4. **Figures S16 – S29.** Visual comparisons of nucleotide sequences of plasmids of three *B. miyamotoi* strains CT13–2396, FR64b and Izh-4 to explore the similarity of plasmids.

**Additional file 3: Figures S30 - S32 and Table S2.** Nucleotide sequences of chromosomes of four *B. miyamotoi* genomes (3-USA, 1-Japan) were aligned to the chromosome of Izh-4 by Mummer and positions of regions containing structural variation were detected by NucDiff and visualized in the IGV browser. **Figure S33**. Similarity of regions which contain the PF57/62 genes located on lp18–1 and lp18–2 plasmids of isolate Izh-4. **Figure S34**. Similarity of regions which contain PF57/62 genes located on lp29 and lp27 plasmids of Izh-4 isolate. **Figure S35**. Alignment of the intergenic region located upstream of the expressed Vmp gene on lp41 of FR64b, Izh-4, CT13–2396, and LB-2001. **Figure S36.** Similarity of the right end of plasmids lp41 and lp23.

**Additional file 4: Figure S37.** PF32 phylogeny. **Figure S38.** PF49 phylogeny. **Figure S39.** PF50 phylogeny. **Figure S40.** PF57/62 phylogeny.

**Additional file 5: Figure S41.** Schematic dot plots of PacBio and ONT contigs with corresponding plasmid names aligned against itself.

## Data Availability

The datasets generated during the current study for Izh-4 isolate are available in the NCBI Sequence Read Archive (SRA) (www.ncbi.nlm.nih.gov/sra/). PacBio raw reads SRR7989200 (https://www.ncbi.nlm.nih.gov/sra/?term=SRR7989200), MinION raw reads SRR7989235 (https://www.ncbi.nlm.nih.gov/sra/?term=SRR7989235), Illumina raw reads of total DNA-library SRR7989238 (https://www.ncbi.nlm.nih.gov/sra/?term=SRR7989238), Illumina raw reads for each PFGE fragments: N1 - SRR7989237 (https://trace.ncbi.nlm.nih.gov/Traces/sra/?run=SRR7989237), N2 - SRR7989232 (https://trace.ncbi.nlm.nih.gov/Traces/sra/?run=SRR7989232), N3 - SRR7989231 (https://trace.ncbi.nlm.nih.gov/Traces/sra/?run=SRR7989231), N4 - SRR7989234 (https://trace.ncbi.nlm.nih.gov/Traces/sra/?run=SRR7989234), N5 - SRR7989233 (https://trace.ncbi.nlm.nih.gov/Traces/sra/?run=SRR7989233), N6 - SRR7989244 (https://trace.ncbi.nlm.nih.gov/Traces/sra/?run=SRR7989244), N7 - SRR7989243 (https://trace.ncbi.nlm.nih.gov/Traces/sra/?run=SRR7989243), N8 - SRR7989198 (https://trace.ncbi.nlm.nih.gov/Traces/sra/?run=SRR7989198), N9 - SRR7989199 (https://trace.ncbi.nlm.nih.gov/Traces/sra/?run=SRR7989199). The final set of chromosome and plasmids for Izn-4 isolate is available in the GenBank: chromosome - CP024390.1 (https://www.ncbi.nlm.nih.gov/nuccore/CP024390), lp72 - CP024391.1 (https://www.ncbi.nlm.nih.gov/nuccore/CP024391), lp70 - CP024392.1 (https://www.ncbi.nlm.nih.gov/nuccore/CP024392.1), lp64 - CP024401.2 (https://www.ncbi.nlm.nih.gov/nuccore/CP024401.2), lp41 - CP024393.1 (https://www.ncbi.nlm.nih.gov/nuccore/CP024393.1), cp30–1 - CP024395.1 (https://www.ncbi.nlm.nih.gov/nuccore/CP024395.1), cp30–2 - CP040828.1 (https://www.ncbi.nlm.nih.gov/nuccore/CP040828.1), lp29 - CP024396.1 (https://www.ncbi.nlm.nih.gov/nuccore/CP024396.1), lp23 - CP024397.1 (https://www.ncbi.nlm.nih.gov/nuccore/CP024397.1), lp27 - CP024398.1 (https://www.ncbi.nlm.nih.gov/nuccore/CP024398.1), lp24 - CP024399.2 (https://www.ncbi.nlm.nih.gov/nuccore/CP024399.2), lp18–2 - CP024400.2 (https://www.ncbi.nlm.nih.gov/nuccore/CP024400.2), lp18–1 - CP024405.2 (https://www.ncbi.nlm.nih.gov/nuccore/CP024405.2), lp13 - CP024404.1 (https://www.ncbi.nlm.nih.gov/nuccore/CP024404.1), lp6 - CP024407.1 (https://www.ncbi.nlm.nih.gov/nuccore/CP024407.1).

## Abbreviations

ANI: Average nucleotide identity
BLAST: Basic local alignment search tool
COG: Clusters of orthologous groups
cp: Circular plasmid
LB: Lyme borreliosis
lp: Linear plasmid
ONT: Oxford nanopore technologies
ORF: Open reading frame
PF: Paralogous gene families of B. burgdorferi
Pfam: Protein family in protein families database (https://pfam.xfam.org/)
PFGE: Pulsed-field gel electrophoresis
RF: Relapsing fever Borrelia
SMRT: Pacific bioscience single molecule real-time technology
SNP: Single nucleotide polymorphisms
Vlp: Variable large proteins
Vmp: Variable major protein
VNTR: Variable number tandem repeats
Vsp: Variable small proteins
