# Supplementary material for: Whole genome sequencing of Borrelia miyamotoi isolate Izh-4: reference for a complex bacterial genome
Source: BMC Genomics. 2020 Jan 6;21:16. doi: 10.1186/s12864-019-6388-4 (PMC6945570; doi:10.1186/s12864-019-6388-4)
Supplement: Supplementary file 2 — Additional file 2: Method S1. Plasmid DNA separation by Pulsed-field Gel Electrophoresis (PFGE). Figures S1 - S6. Visual comparisons of ONT, PacBio and final assemblies of plasmids of Borrelia miyamotoi isolate Izh-4. Figures S7 - S15. Visual comparisons of contigs assembled from short Illumina reads for each PFGE fragment and final assemblies of plasmids of Borrelia miyamotoi isolate Izh-4. Figures S16 – S29. Visual comparisons of nucleotide sequences of plasmids of three B. miyamotoi strains CT13–2396, FR64b and Izh-4 to explore the similarity of plasmids. [file 12864_2019_6388_MOESM2_ESM.docx]

**Supplementary materials**

**Supplementary Method 1. Plasmid DNA separation by Pulsed-field Gel Electrophoresis (PFGE).**

Agarose plugs for particular strains were prepared from 100 µl cell suspension contained appx. 10^9^ cell, 7 µl Proteinase K (20 mg/ml) and 100 µl of 1% SeakemGold Agarose (Lonza, USA). The agarose plug was incubated in 5 ml of cell lysis buffer/Proteinase K mixture (50 mM Tris, 50 mM EDTA, pH 8.0 and 1% Lauryl Sarkosyl /25 µl Proteinase K (20 mg/ml) ) for 2 hours in a shaking water bath at 54^0^C. Following the lysis step, plugs were washed twice in deionized water and three times in TE-buffer.

We used 1% Low Melt Agarose (#1613111, BioRad, USA) in 0.5X Tris-Borate EDTA Buffer (TBE) for separation of plasmids using the CHEF Mapper XA System (Biorad, USA). To obtain good resolution between 30 and 90 kb as well as between 5 and 50 kb electrophoresis conditions were optimized empirically as follows: state #1 - 27 hours, angle 120^0^, switch time from 3 sec to 8 sec, ramping factor "-1.002"; state #2 - 20.5 hours, forward voltage gradient 9V/cm, switch time from 0.11 sec to 0.92 sec, ramping factor was linear, then reverse voltage gradient 6 V/cm, switch time from 0.11 sec to 0.92 sec, ramping factor was linear. The buffer temperature was maintained at 14^0^С.

The agarose gel was stained with ethidium bromide (8 µg/ml) for 30 minutes and washed twice in deionized water (20 minutes each). PFGE images, generated using a GelDocXR system (BioRad), were analyzed in Bionumerics v6.6 (AppliedMath, Belgium). Concatemers of phage λcl857Sam7 (# 1703635, BioRad) and Lambda-HindIII were used as size ladders.

**Supplemental Figures S1 - S6. Visual comparisons of ONT, PacBio and final assemblies of plasmids of *Borrelia miyamotoi* isolate Izh-4.**

Supplemental Figure S1. Misassembled ONT tig00009030 consisted of two plasmids, lp24 and lp27. Visual comparisons of ONT, PacBio and final assemblies of plasmids of *B. miyamotoi* isolate Izh-4. Black blocks indicates raw MinION (ONT) contigs, red blocks indicates raw PacBio contigs, and green blocks - final set of validated contigs. Orange chords - indicate regions of similarity >5 kb. Contigs characterized by long reads with a low coverage (2-3x) and which had a high indentity level (95%) to other contigs were removed from further analysis and are indicated by red asterisks.


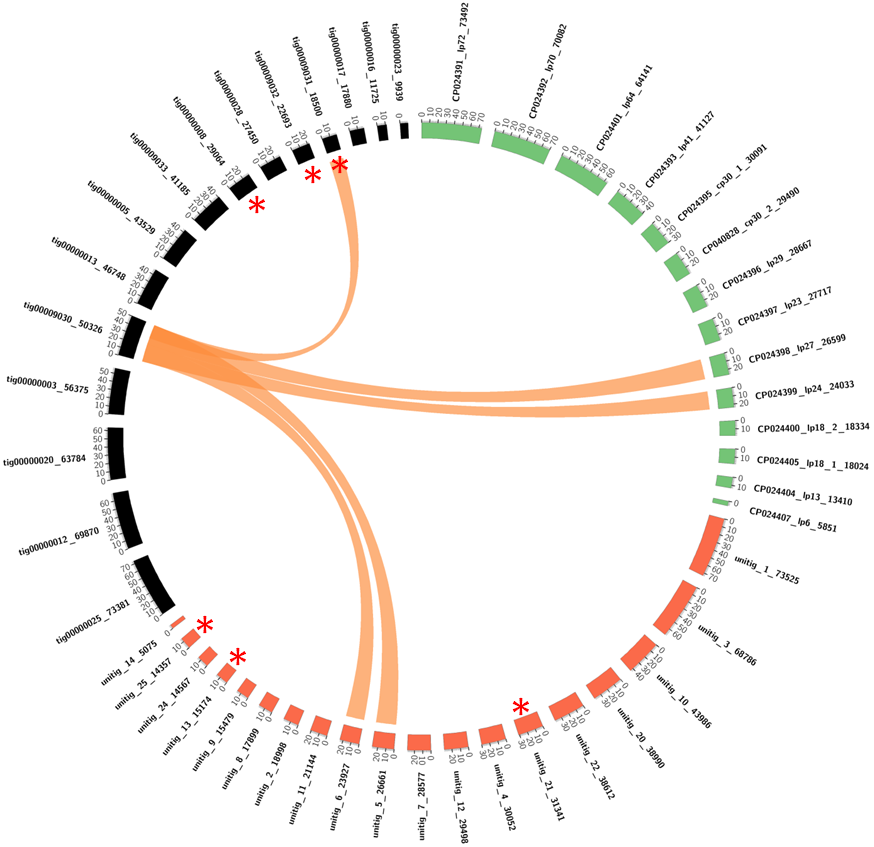


Supplemental Figure S2. Misassembled ONT tig00000013 consisted of two plasmids, lp18 and lp29. Visual comparisons of ONT, PacBio and final assemblies of plasmids of *Bm* isolate Izh-4. Black blocks indicates raw MinION (ONT) contigs, red blocks indicates raw PacBio contigs, and green blocks - final set of validated contigs. Orange chords - indicate regions of similarity >5 kb. Contigs characterized by long reads with a low coverage (2-3x) and which had a high identity level (95%) to other contigs were removed from further analysis and are indicated by red asterisks.


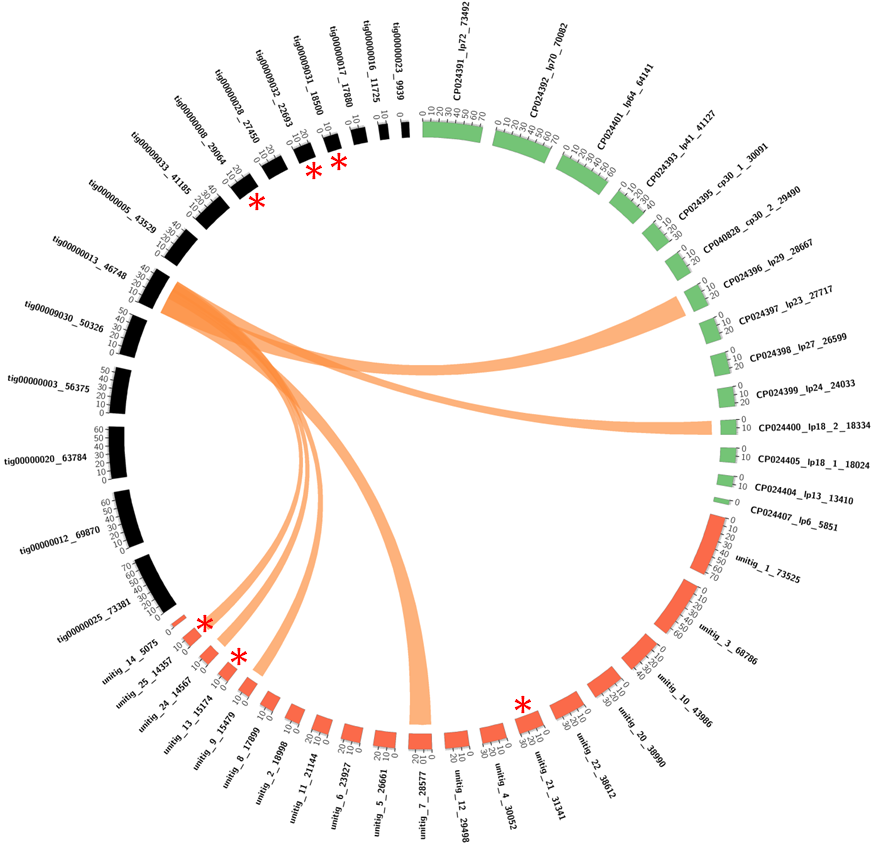


Supplemental Figure S3. Example of artificial merging of two plasmids. tig00000013 represented two plasmids that were artificially merged by the Canu assembler. Mapped corrected ONT reads to tig00000013 were visualized in CLC Genomic Workbench.


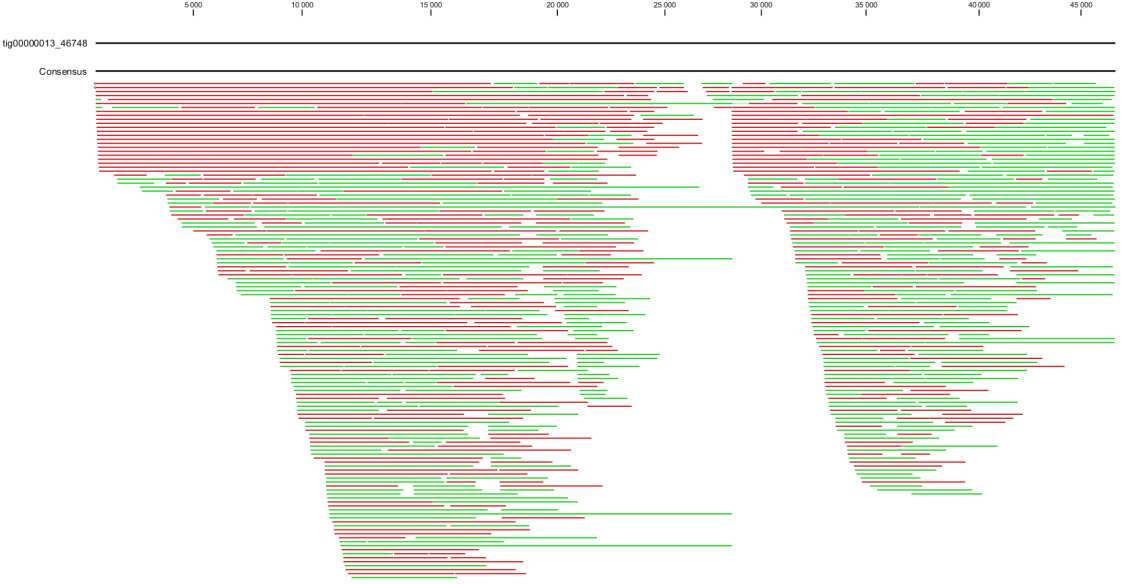


Supplemental Figure S4. Misassembled unitig #3 of PacBio assembly. Visual comparisons of ONT, PacBio and final assemblies of plasmids of *Bm* isolate Izh-4. Black blocks indicates raw MinION (ONT) contigs, red blocks indicates raw PacBio contigs, and green blocks - final set of validated contigs. Orange chords - indicate regions of similarity >5 kb. Contigs characterized by long reads with a low coverage (2-3x) and which had a high identity level (95%) to other contigs were removed from further analysis and are indicated by red asterisks.


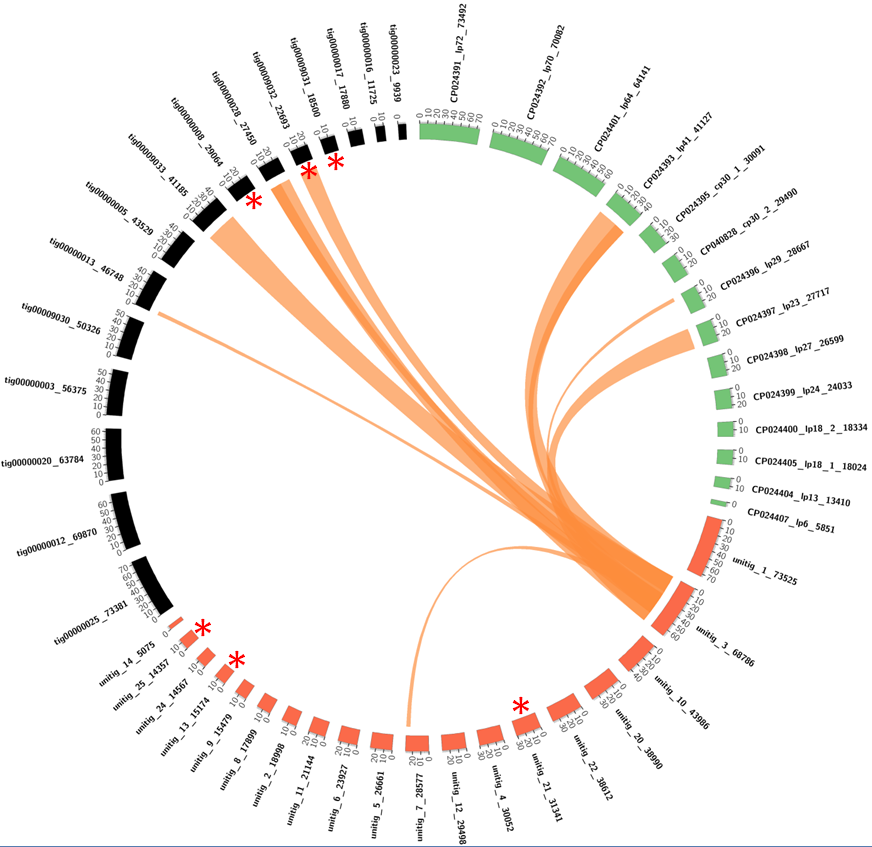


*

*

*

Supplemental Figure S5. ONT contig 70kb was mistakenly separated in to three PacBio contigs (unitigs #20, and #22 and #2). Visual comparisons of ONT, PacBio and final assemblies of plasmids of *Bm* isolate Izh-4. Black blocks indicates raw MinION (ONT) contigs, red blocks indicates raw PacBio contigs, and green blocks - final set of validated contigs. Orange chords - indicate regions of similarity >5 kb. Contigs characterized by long reads with a low coverage (2-3x) and which had a high identity level (95%) to other contigs were removed from further analysis and are indicated by red asterisks.


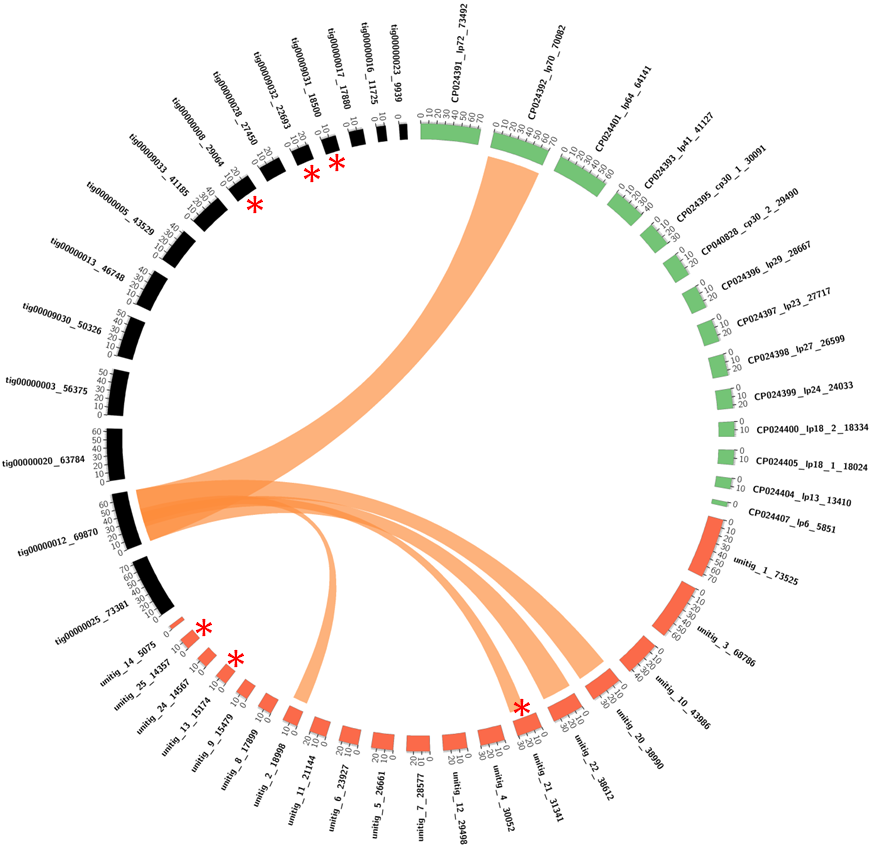


Supplemental Figure S6. 64kb ONT contig is partially represented in unitig#10. Visual comparisons of ONT, PacBio and final assemblies of plasmids of *Bm* isolate Izh-4. Black blocks indicates raw MinION (ONT) contigs, red blocks indicates raw PacBio contigs, and green blocks - final set of validated contigs. Orange chords - indicate regions of similarity >5 kb. Contigs characterized by long reads with a low coverage (2-3x) and which had a high identity level (95%) to other contigs which were removed from further analysis and are indicated by red asterisks.


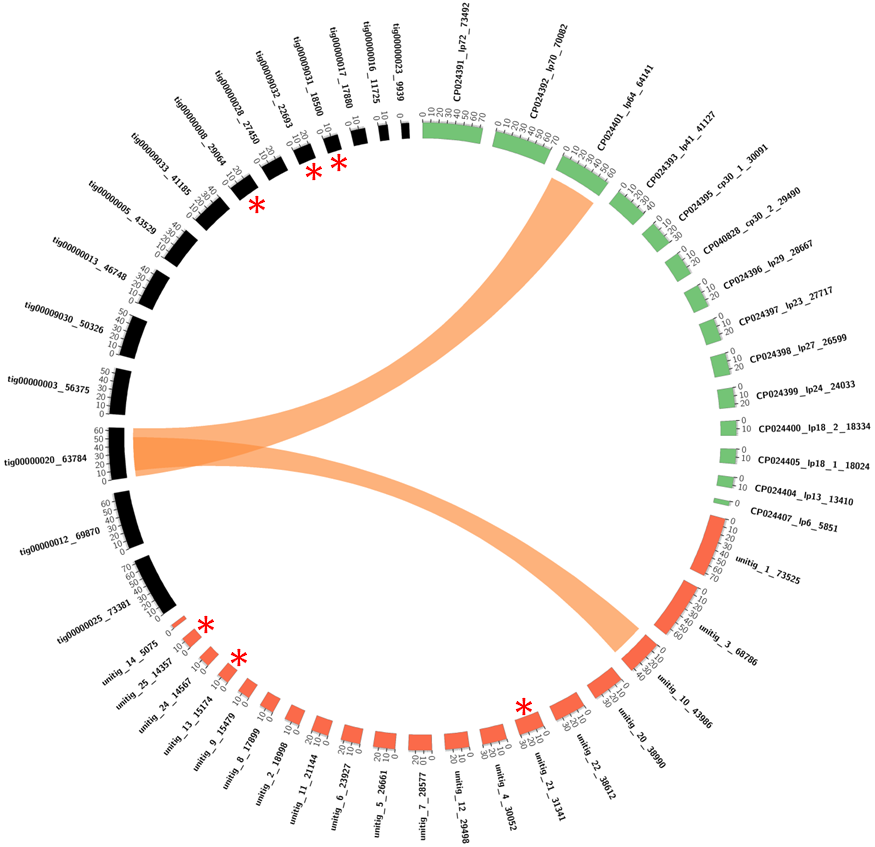


**Supplemental Figures S7 – S15. Visual comparisons of contigs assembled from short Illumina reads for each PFGE fragment and final assemblies of plasmids of Izh-4 *Borrelia miyamotoi* strain.**

PFGE-fragments are numbered according to Figure 1. Red blocks indicate the final assembly of isolate Izh-4, green blocks refer to assembled contigs from the PFGE fragment. Orange chords - regions of similarity (>90%) with > 5 kb in size and green chords - regions of similarity that were <5 kb.

Supplemental Figure S7. For the PFGE fragment N1 85 contigs were assembled from Illumina short reads, but only one contig of a length of 72,707 bp completely reproduced the lp72 plasmid in the final assembly.


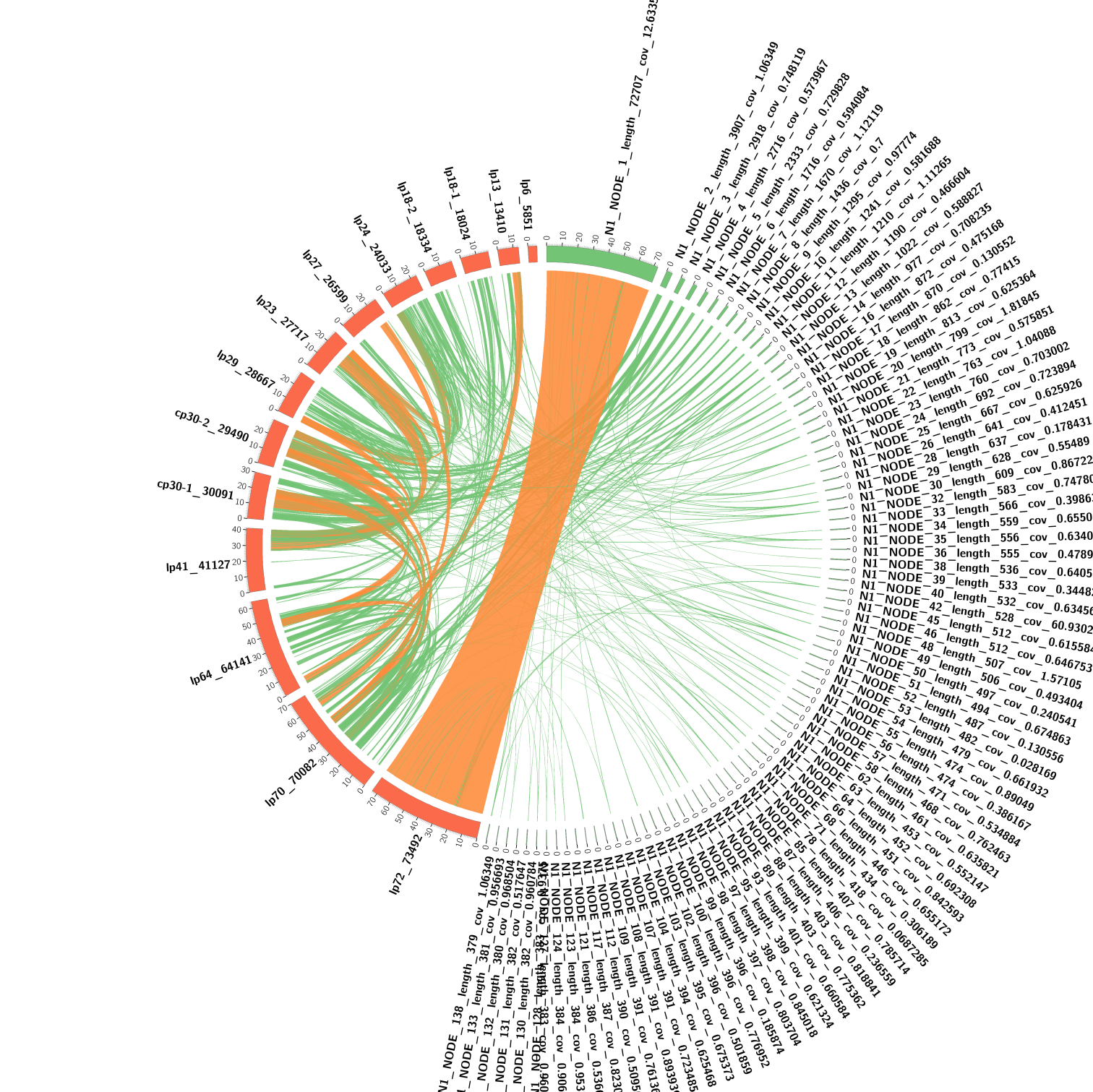


Supplemental Figure S8. For the PFGE fragment N2 128 contigs were collected but only 4 contigs with a length of 11 - 20 kb were related to plasmid lp70.


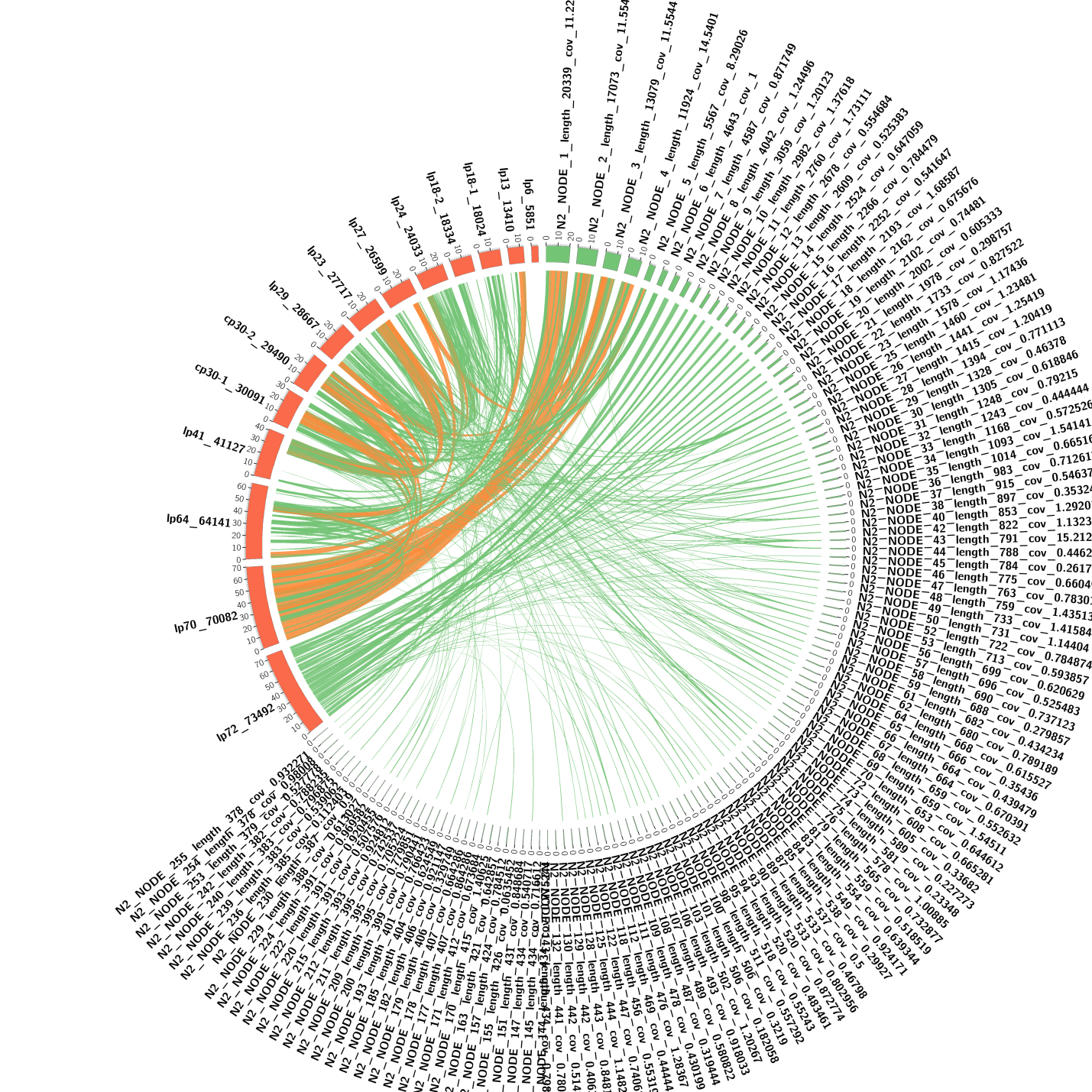


Supplemental Figure S9. For the PFGE fragment N3 141 contigs were collected, while only 3 contigs with a length of 11-15 kb mapped with plasmid lp64.


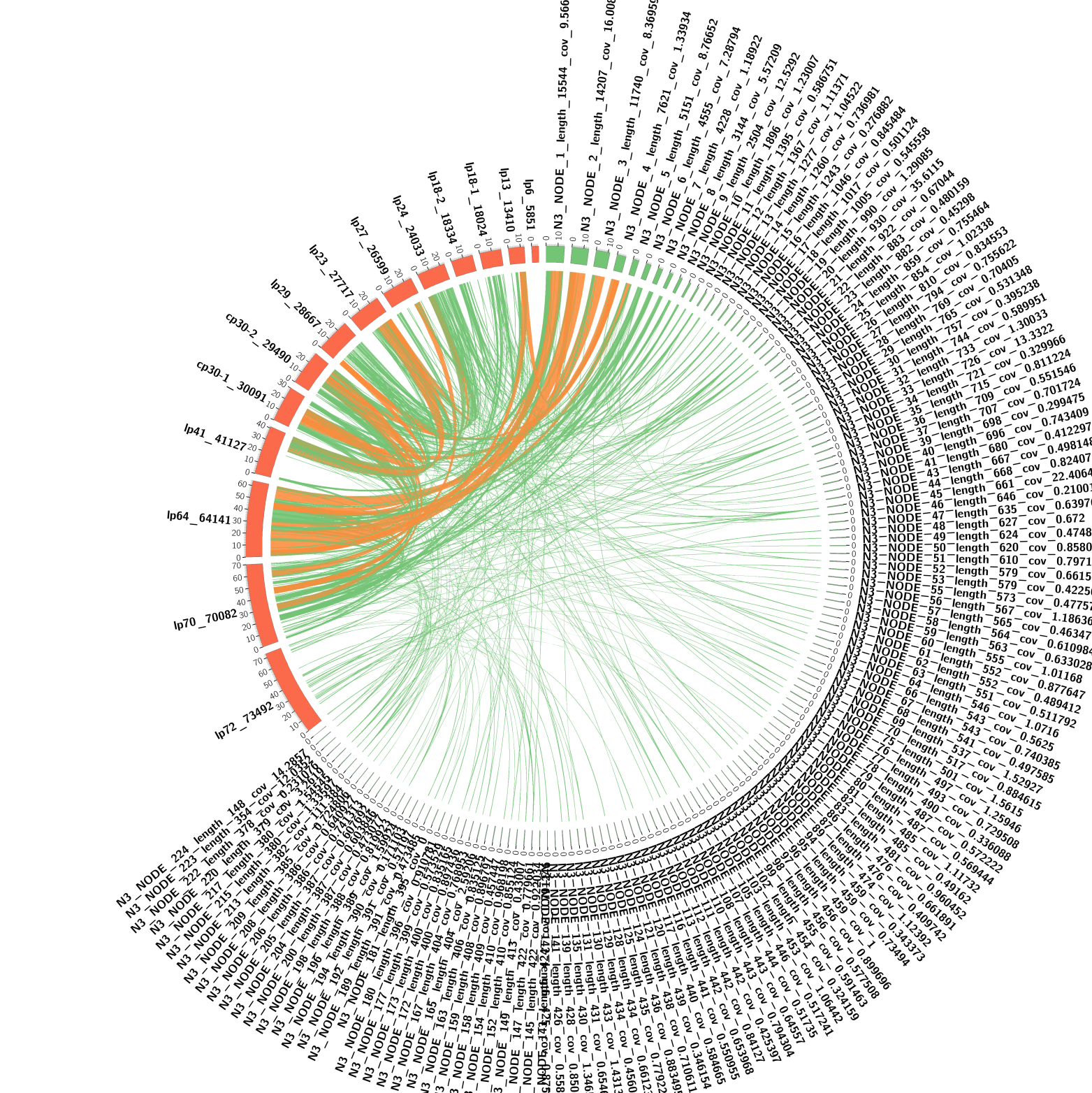


Supplemental Figure S10. For the PFGE fragment N4 100 contigs were collected, while only one contig with a length of 41 kb reproduced the plasmid lp41.


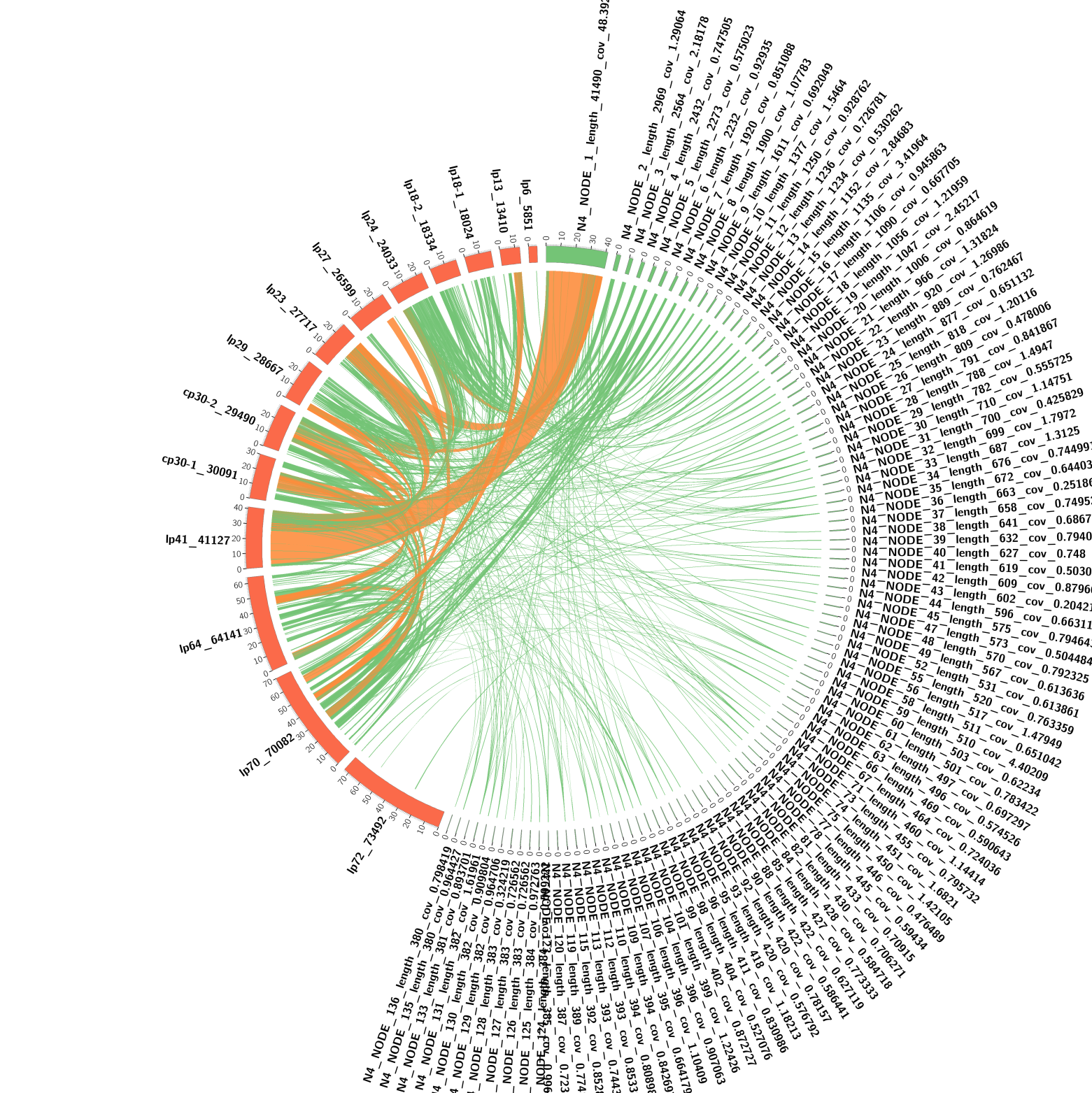


Supplemental Figure S11. For the PFGE fragment N5 66 contigs were collected, while only one contig with a length of 28 kb reproduced the plasmid lp29.


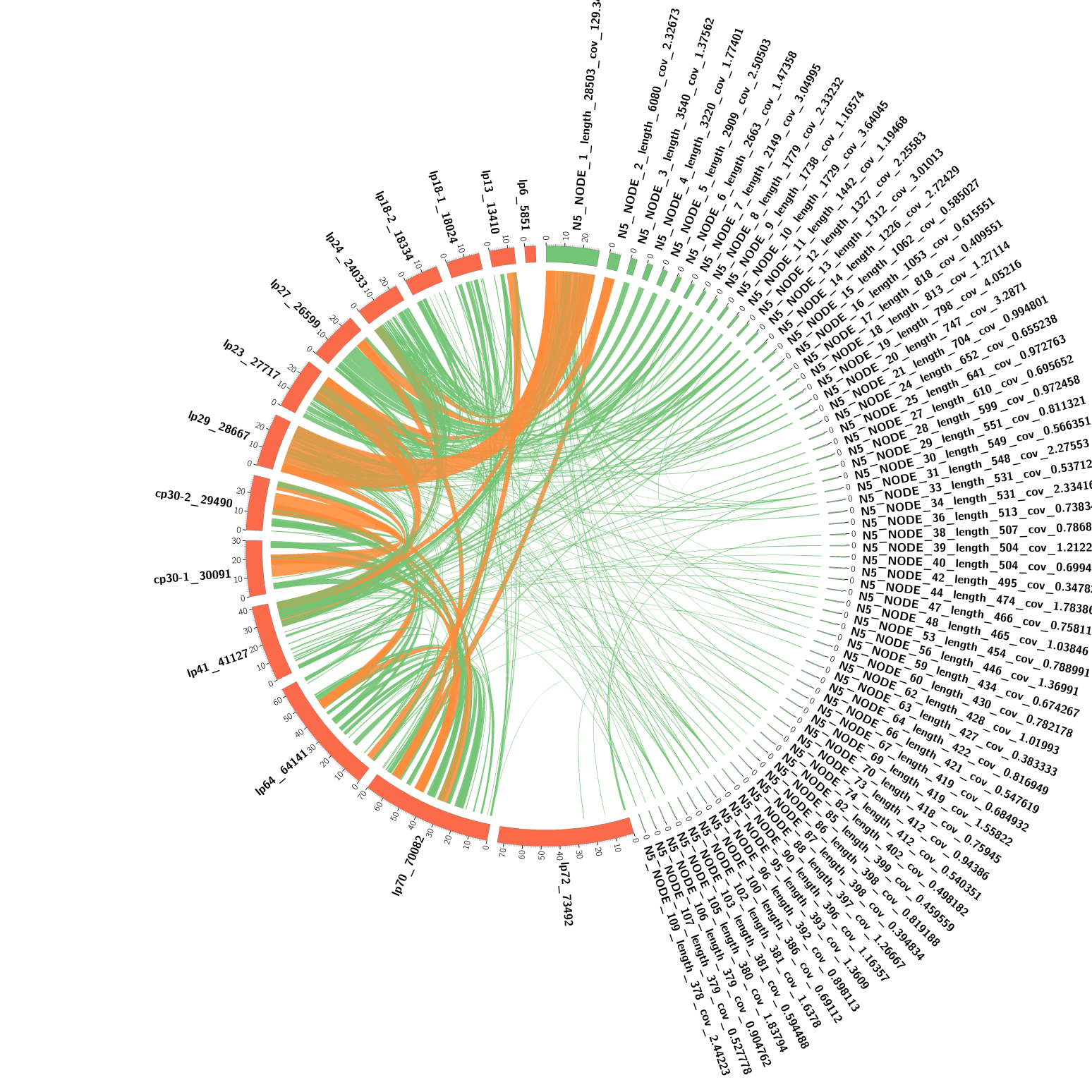


Supplemental Figure S12. 41 contigs were collected for PFGE fragment N6, while two contigs 27 kb and 26 kb in length reproduced two separate plasmids lp23 and lp27.


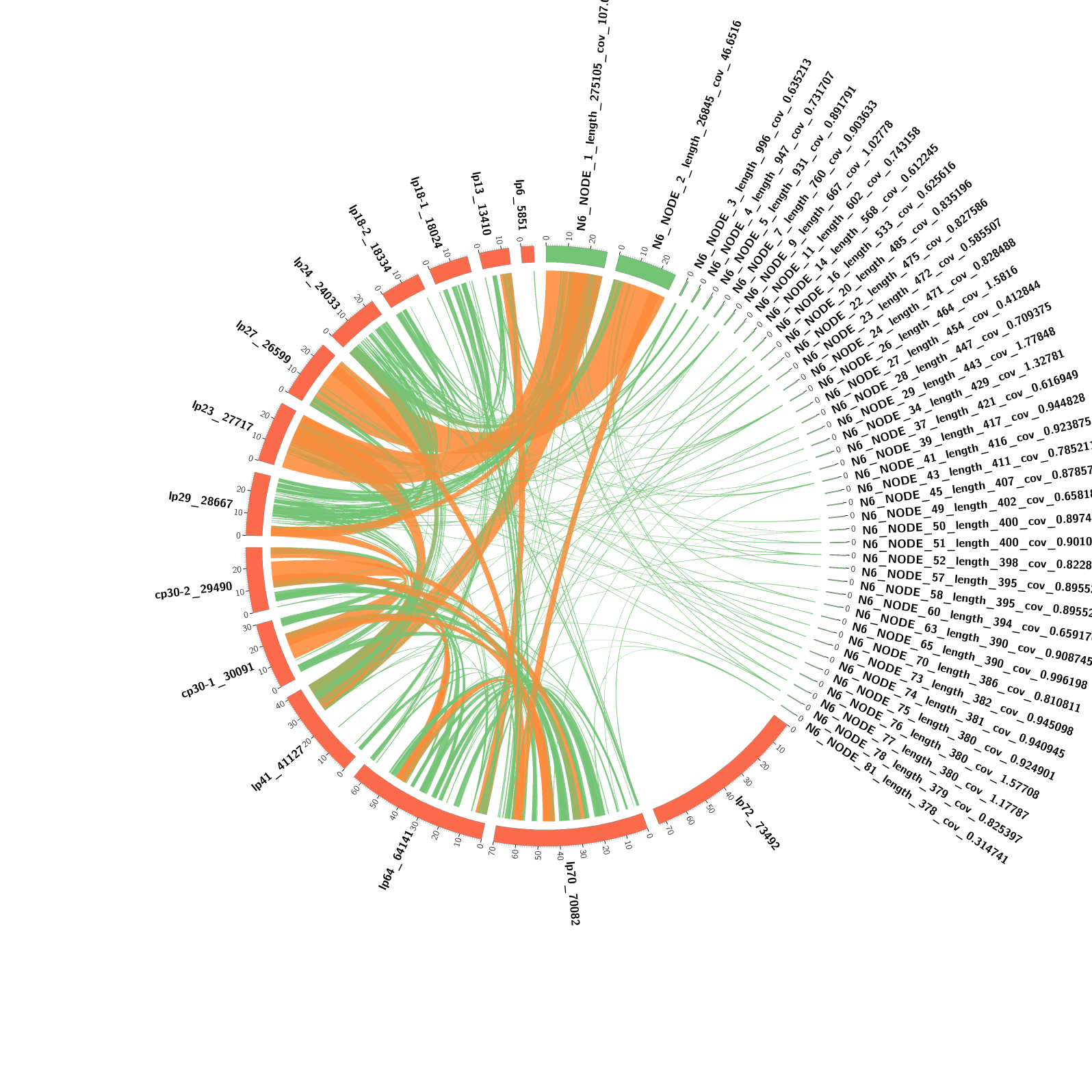


Supplemental Figure S13. 53 contigs were collected for PFGE fragment N7, and only one contig with a length of 24 kb reproduced the plasmid lp24.


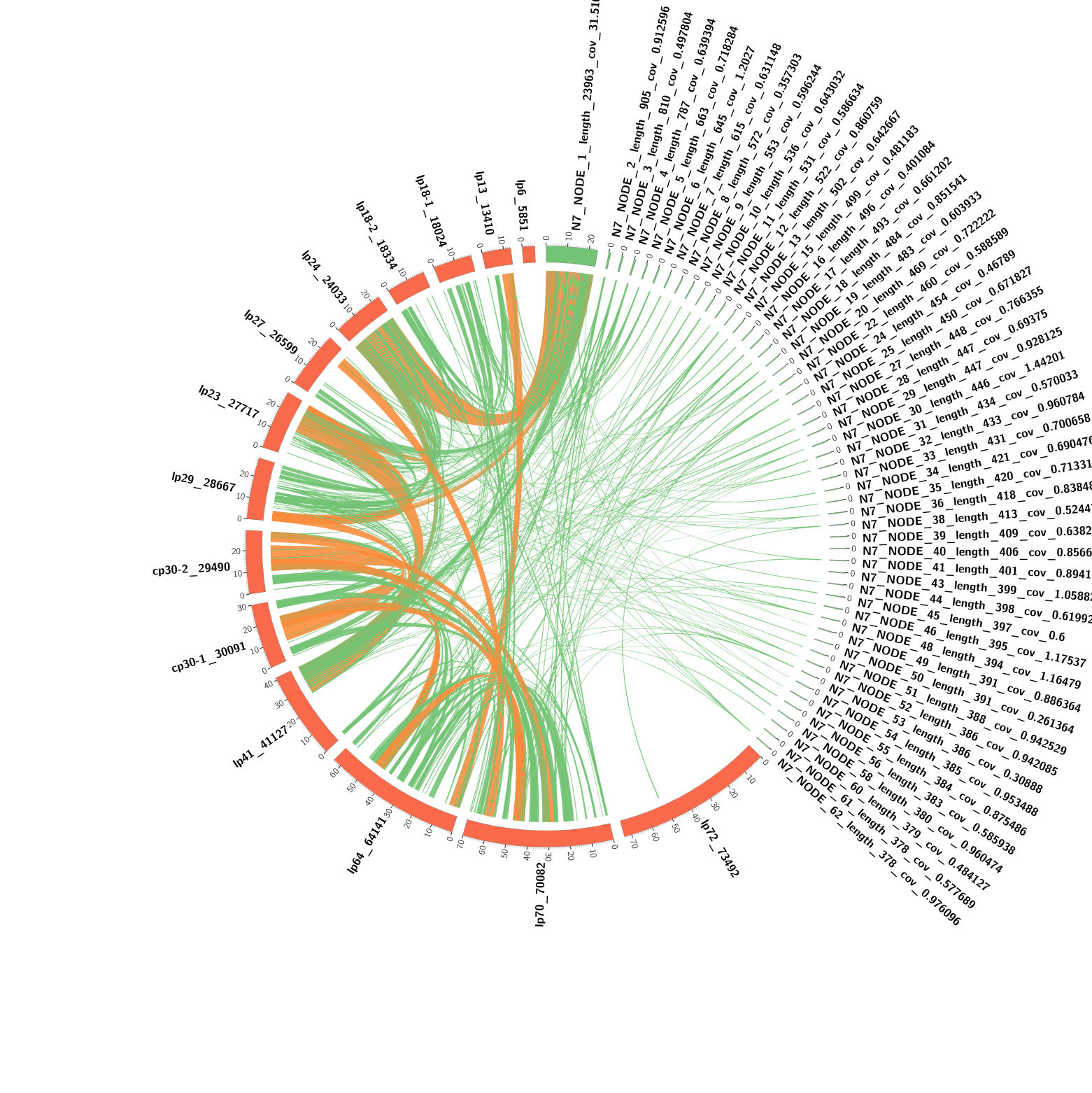


Supplemental Figure S14. For the PFGE fragment N8, 22 contigs were assembled, with one contig reproducing the lp18-2 plasmid, and three other contigs partially reproducing the lp18-1 plasmid.


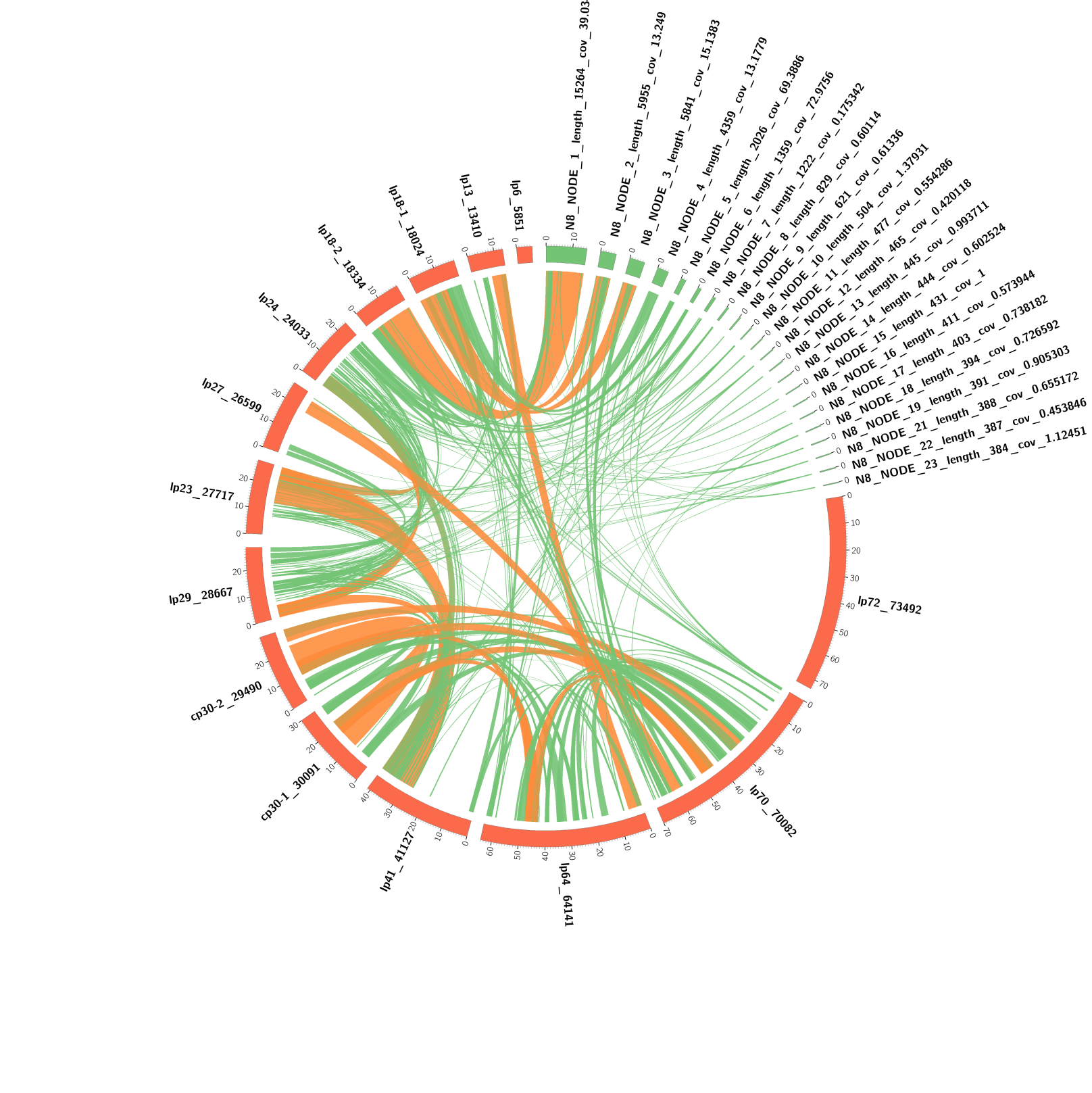


Supplemental Figure S15. Twelve contigs were collected for the PFGE fragment N9, with one contig reproducing the lp13 plasmid and the other single contig reproducing the plasmid lp6.


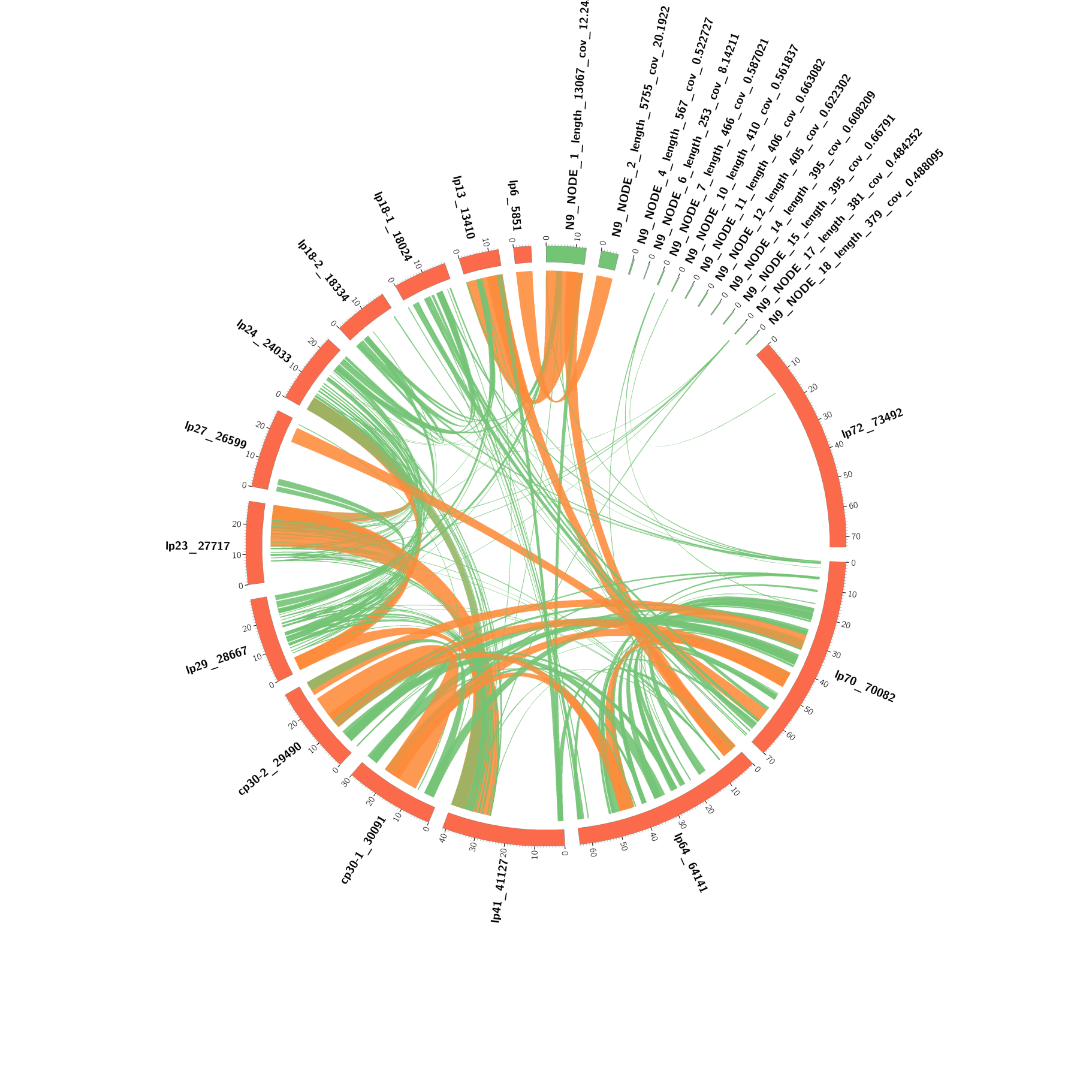


**Supplemental Figures S16 – S29. Visual comparisons of nucleotide sequences of plasmids of three *B. miyamotoi* strains CT13-2396 (green blocks), FR64b (black blocks) and Izh-4 (red blocks) to explore the similarity of plasmids.**

Orange chords - the regions of similarity (> 90%) > 5 kb in length and green chords - the regions of similarity (>90%) < 5 kb. In the analysis, it must be borne in mind that most contigs smaller than 15 kb in size and associated with the FR64b genome, are not complete plasmids, but are parts of larger plasmid sequences.

Supplemental Figure S16. Izh-4 lp72 is similar to analogous plasmids in CT13-2396 and FR64b.


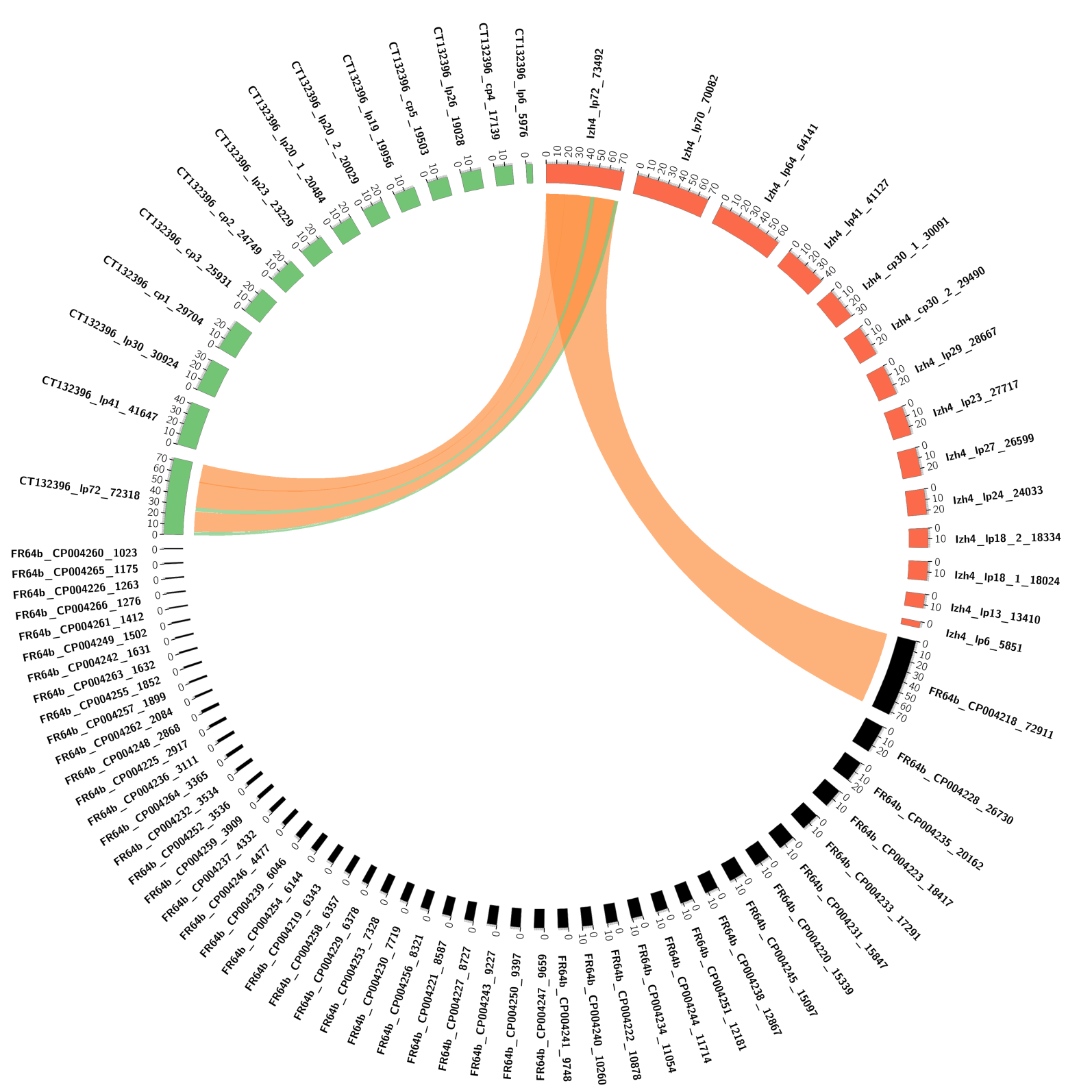


Supplemental Figure S17. Izh-4 lp70 is similar to several separate contigs in FR64b.


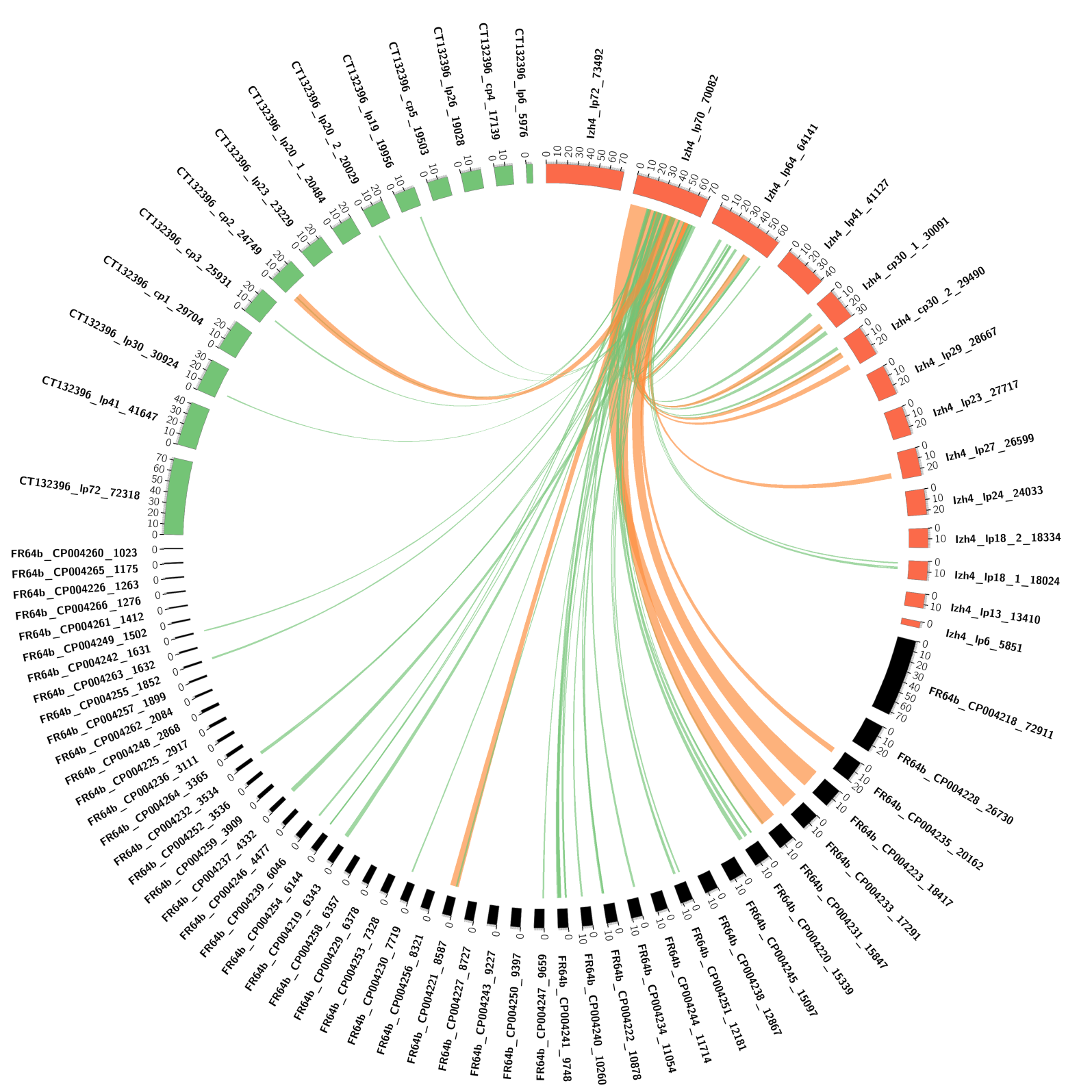


Supplemental Figure S18. Izh-4 lp64 is similar to several separate contigs in FR64b.


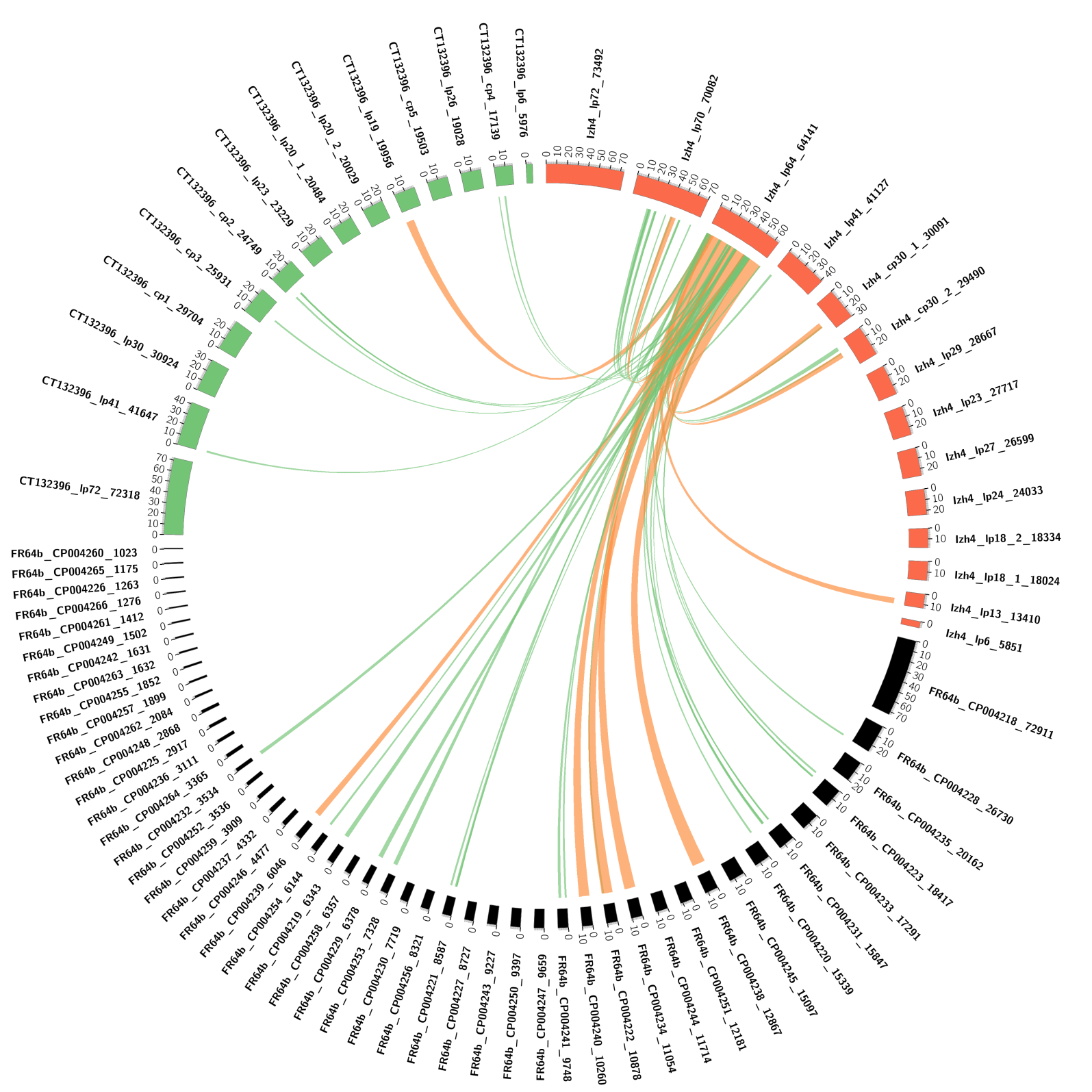


Supplemental Figure S19. Izh-4 lp41 is in part similar to lp41 in CT13-2396 and two contigs in FR64b.


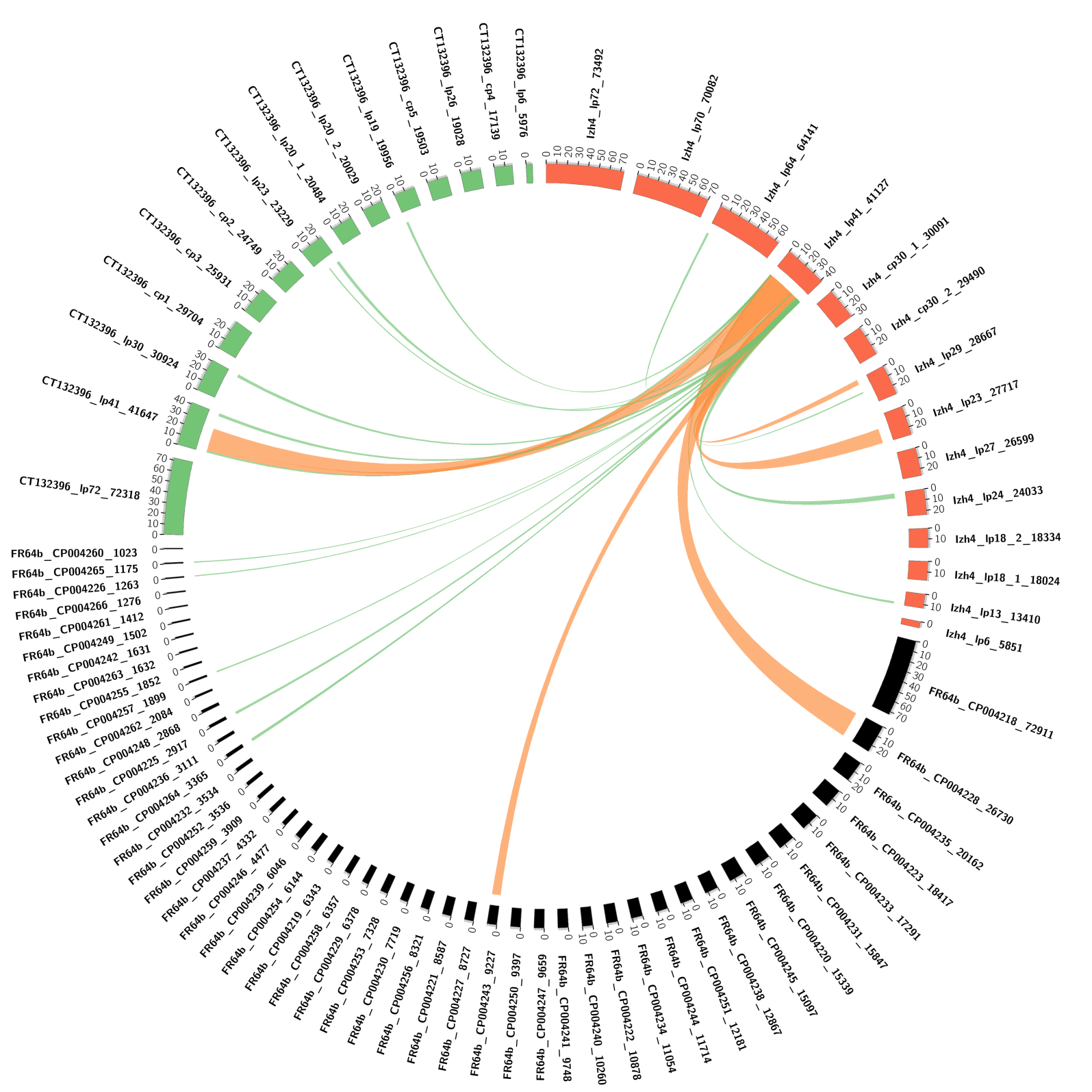


Supplemental Figure S20. Izh-4 cp30-1 is similar to Izh-4 cp30-2, several contigs in FR64b and absent in CT13-2396.


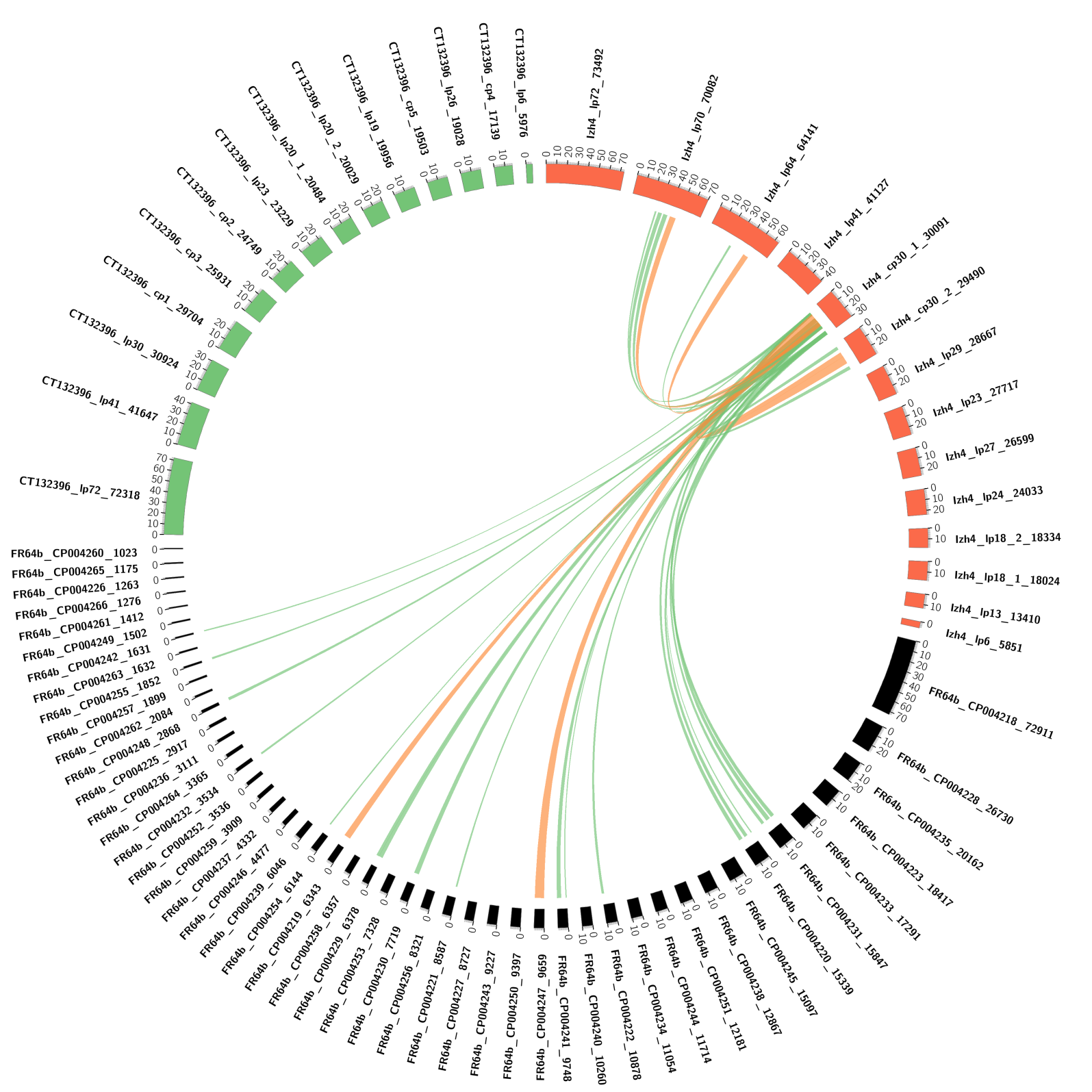


Supplemental Figure S21. Izh-4 cp30-2 is similar to Izh-4 cp30-1, several contigs in FR64b and absent in CT13-2396.


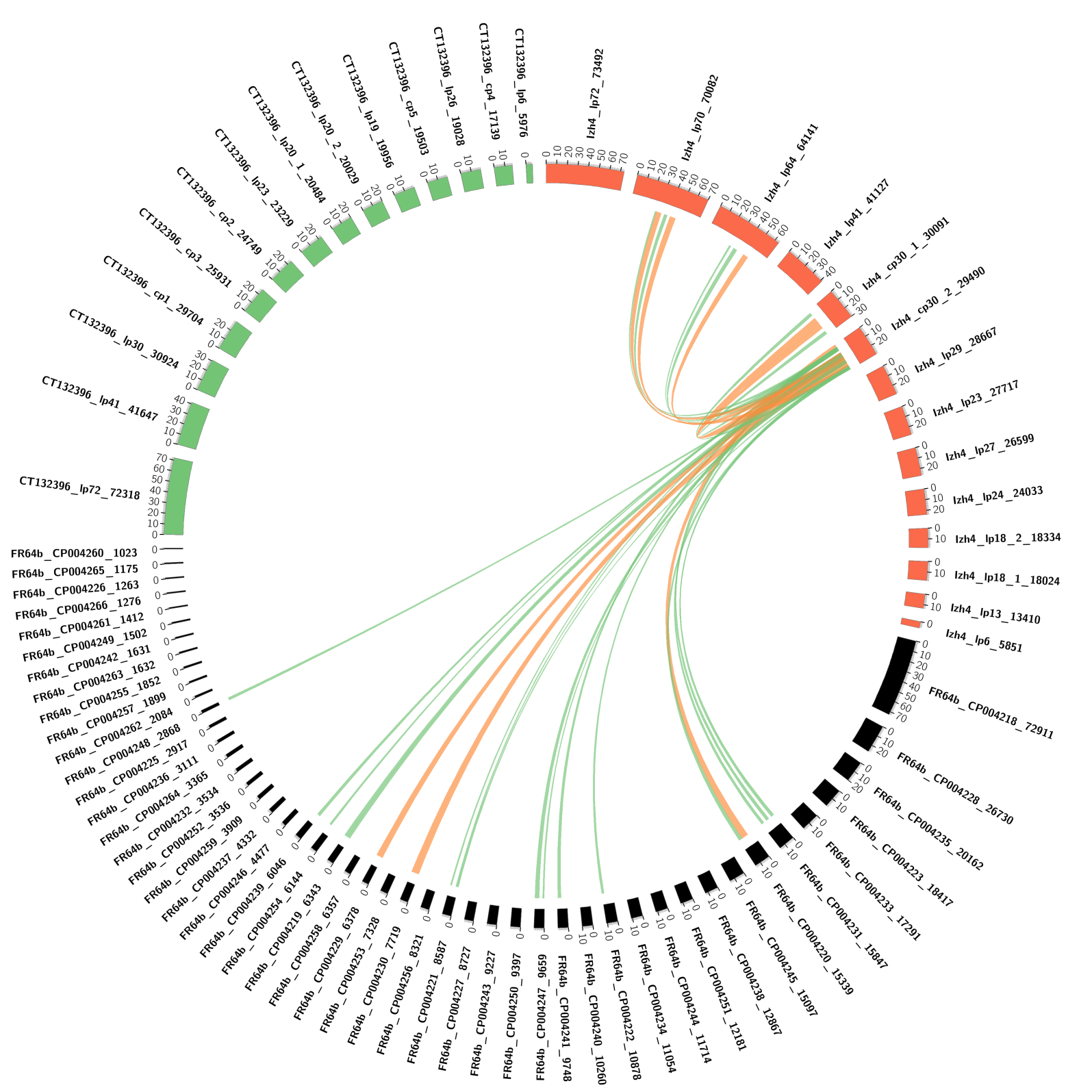


Supplemental Figure S22. Izh-4 lp29 is in part similar to two small contigs in FR64b.


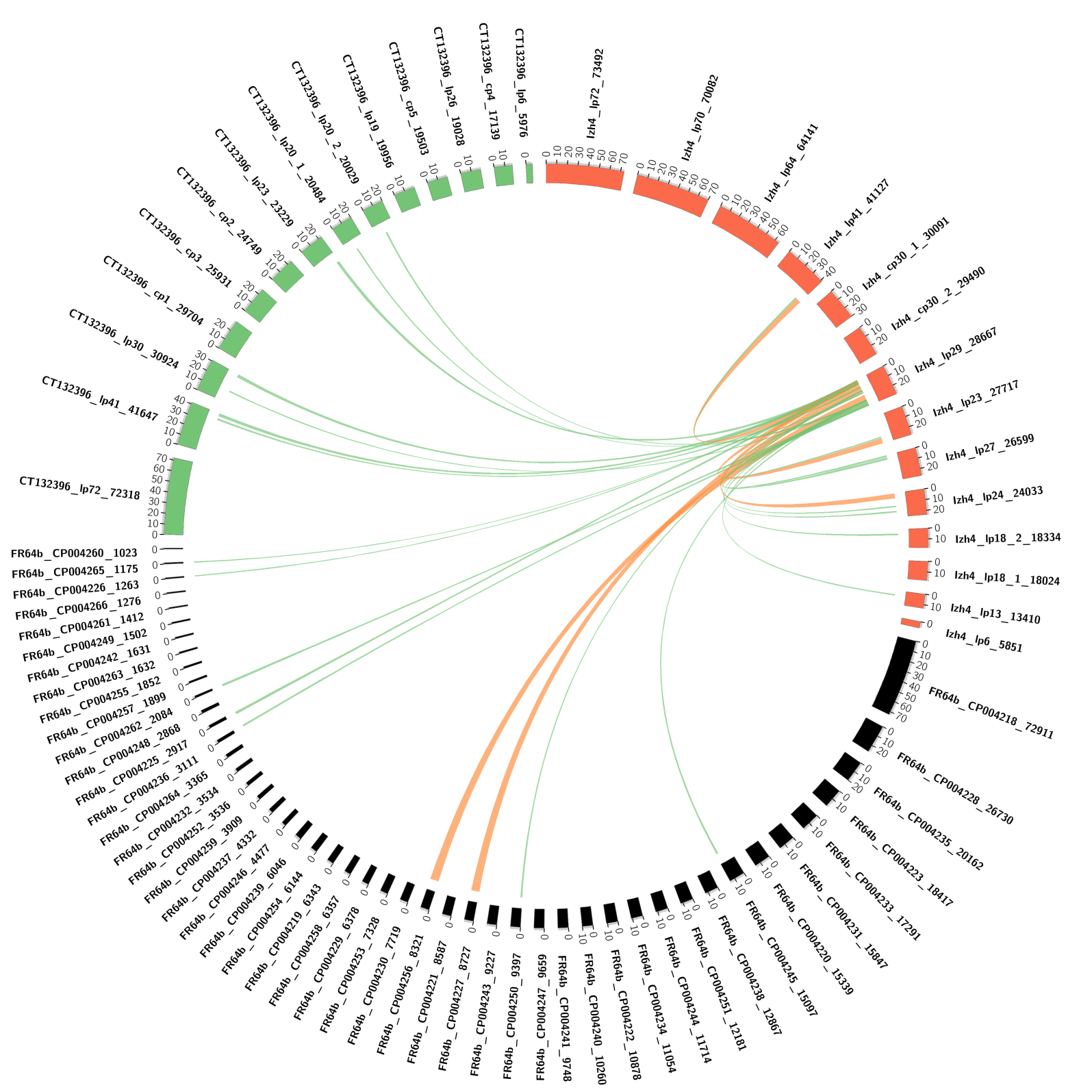


Supplemental Figure S23. Izh-4 lp23 is in part similar to lp23 in CT13-2396 and to two small contigs in FR64b.


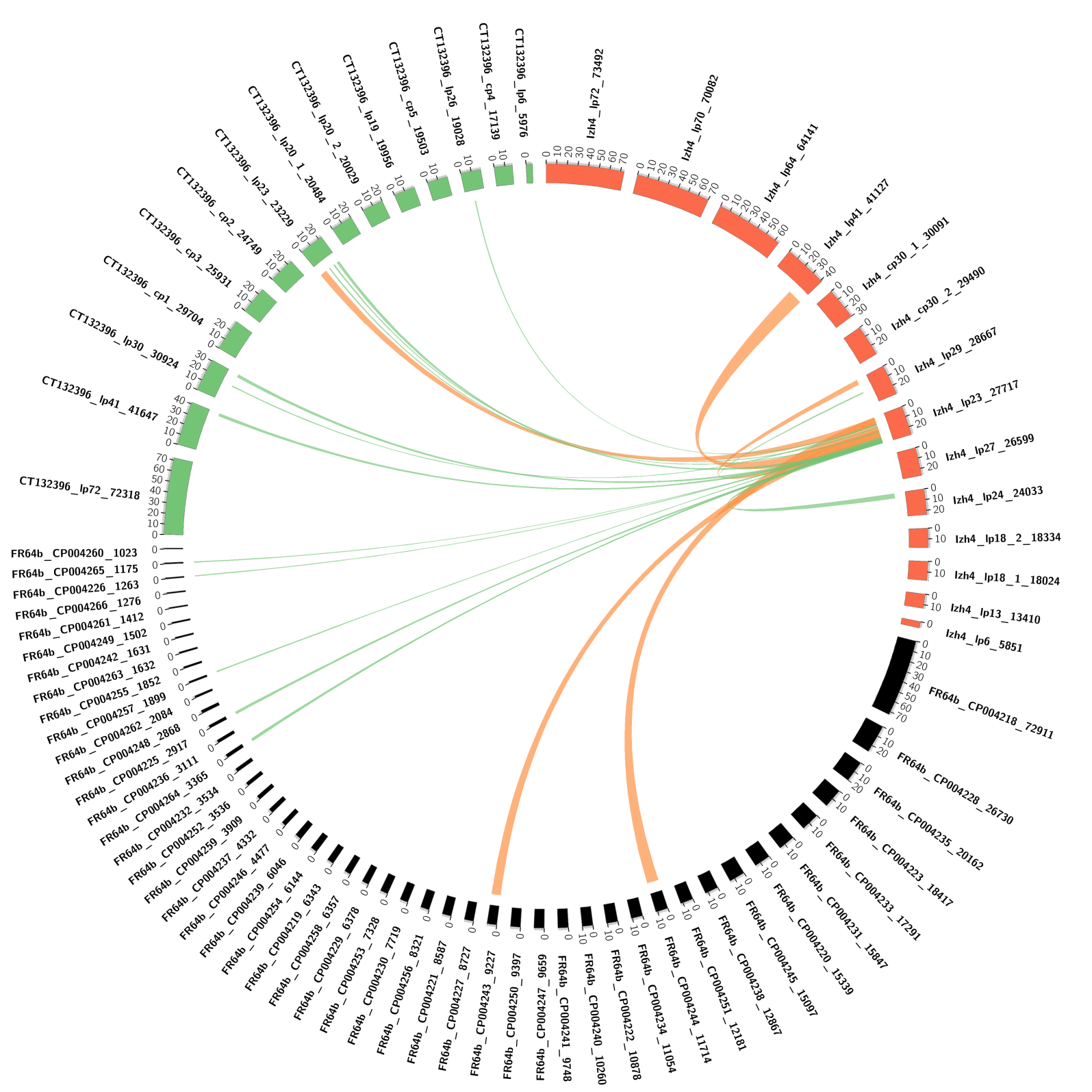


Supplemental Figure S24. Izh-4 lp27 is absent in CT13-2396 and similar to one contig in FR64b.


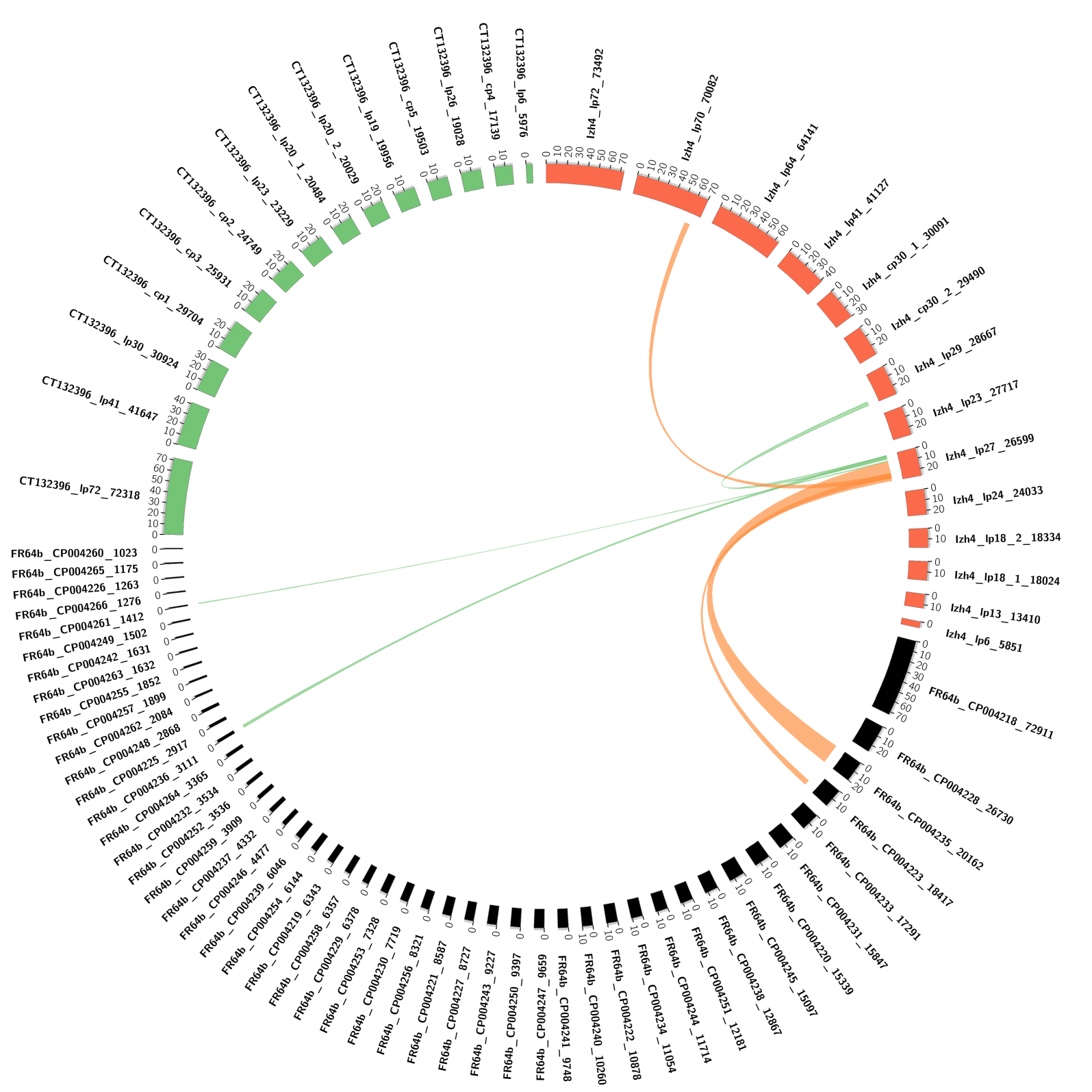


Supplemental Figure S25. Izh-4 lp24 is absent in CT13-2396 and in part similar to one contig in FR64b.


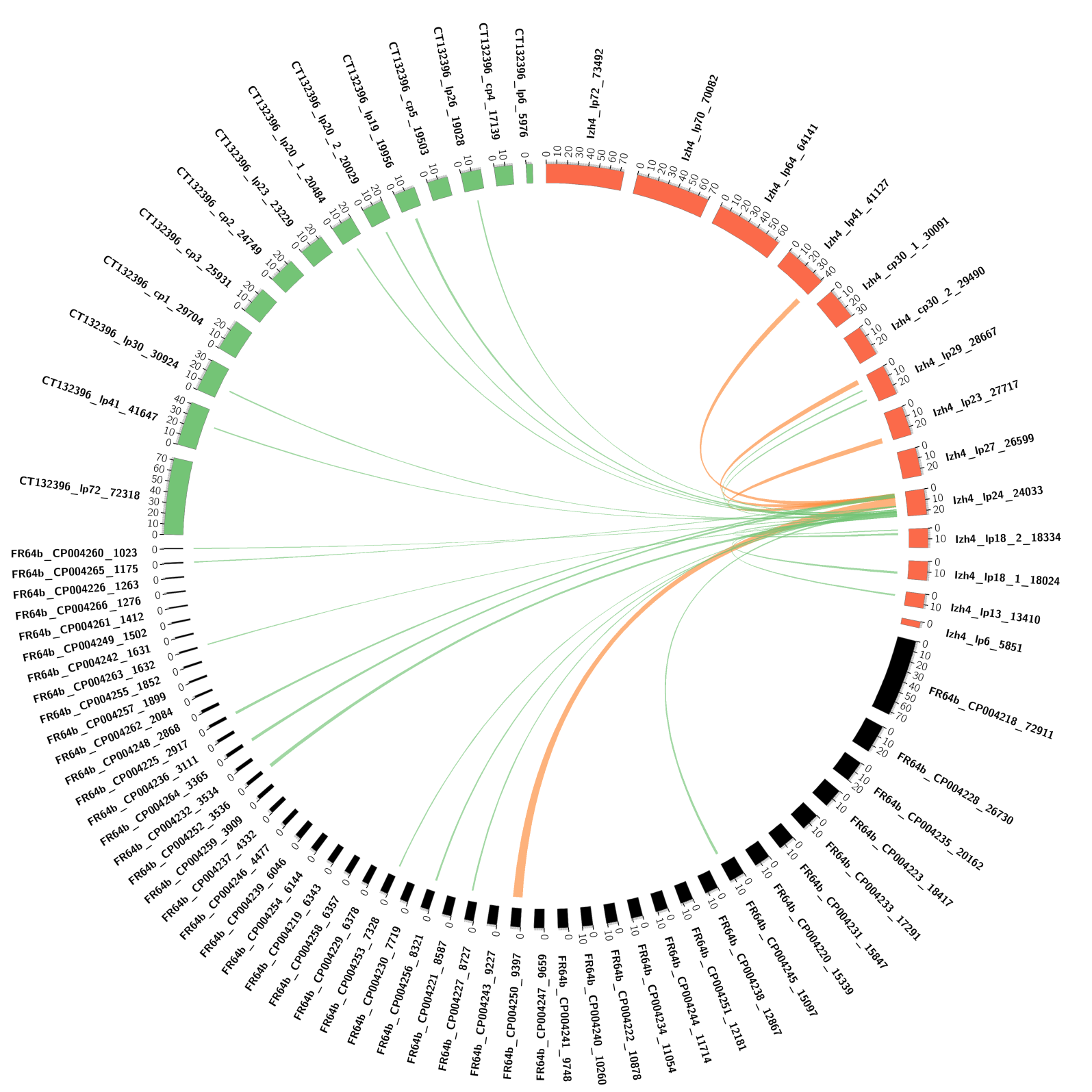


Supplemental Figure S26. Izh-4 lp18-2 is similar to one contig in FR64b and is related to lp20-1 in CT13-2396.


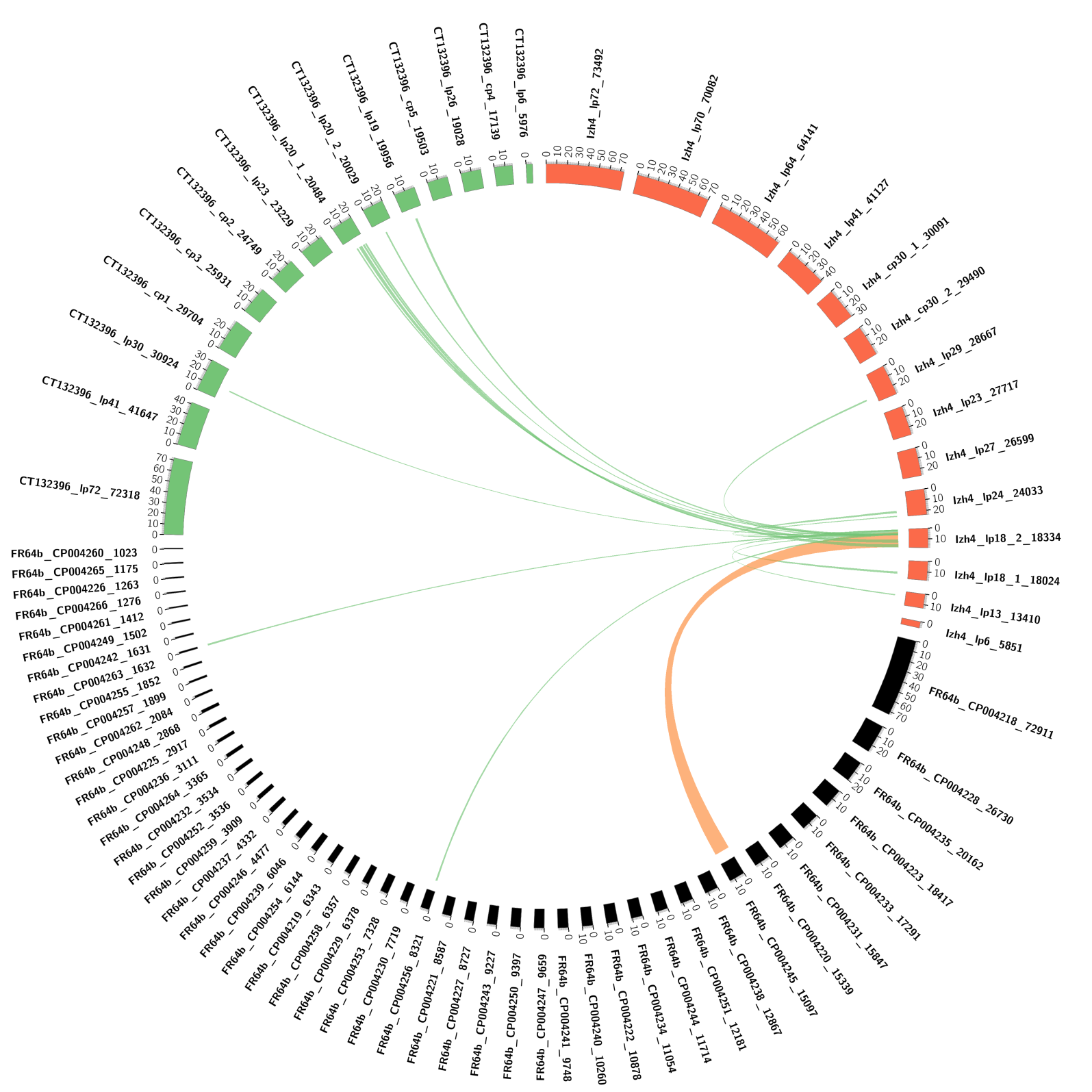


Supplemental Figure S27. Izh-4 lp18-1 is similar to one contig in FR64b.


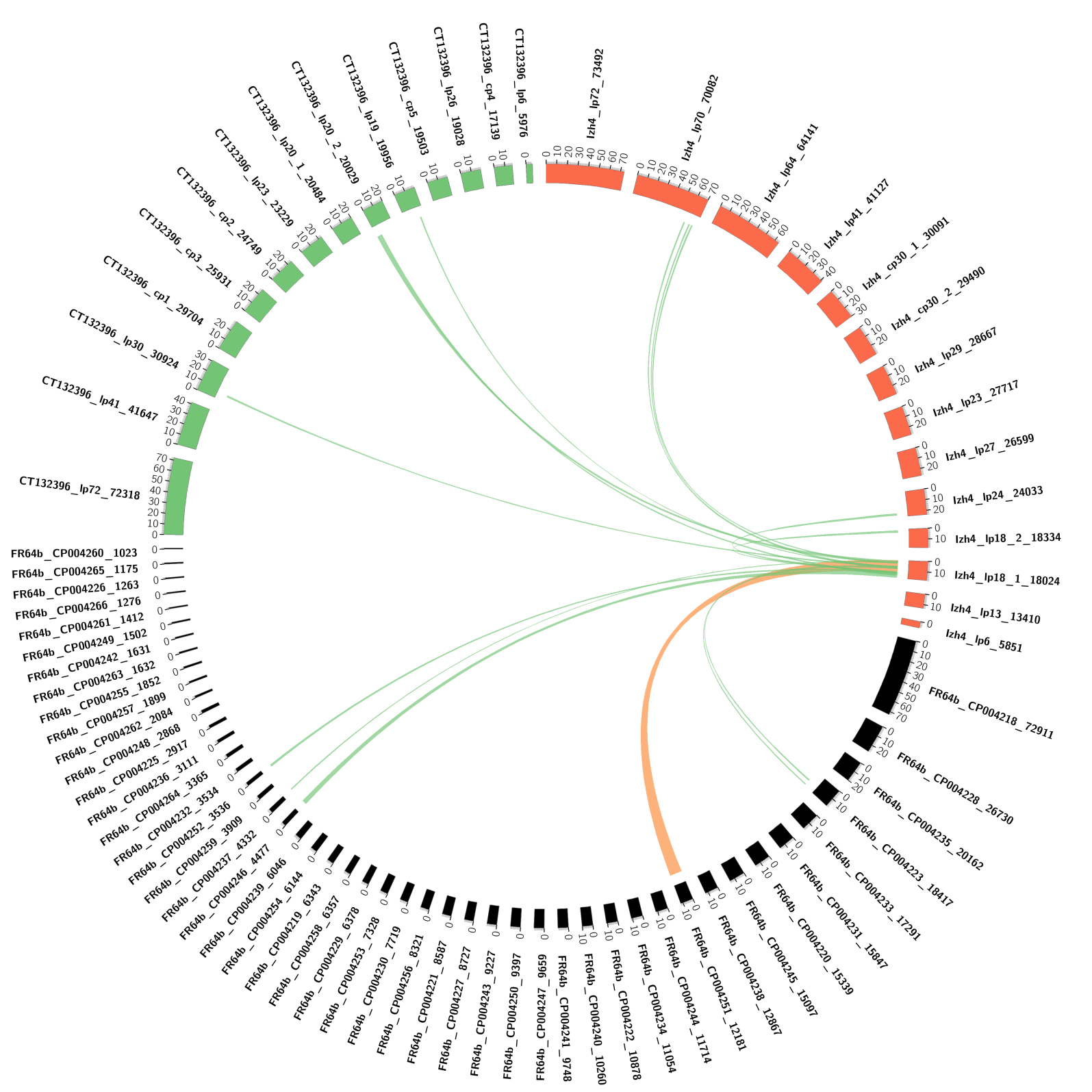


Supplemental Figure 28. Izh-4 lp13 is in part similar to one contig in FR64b.


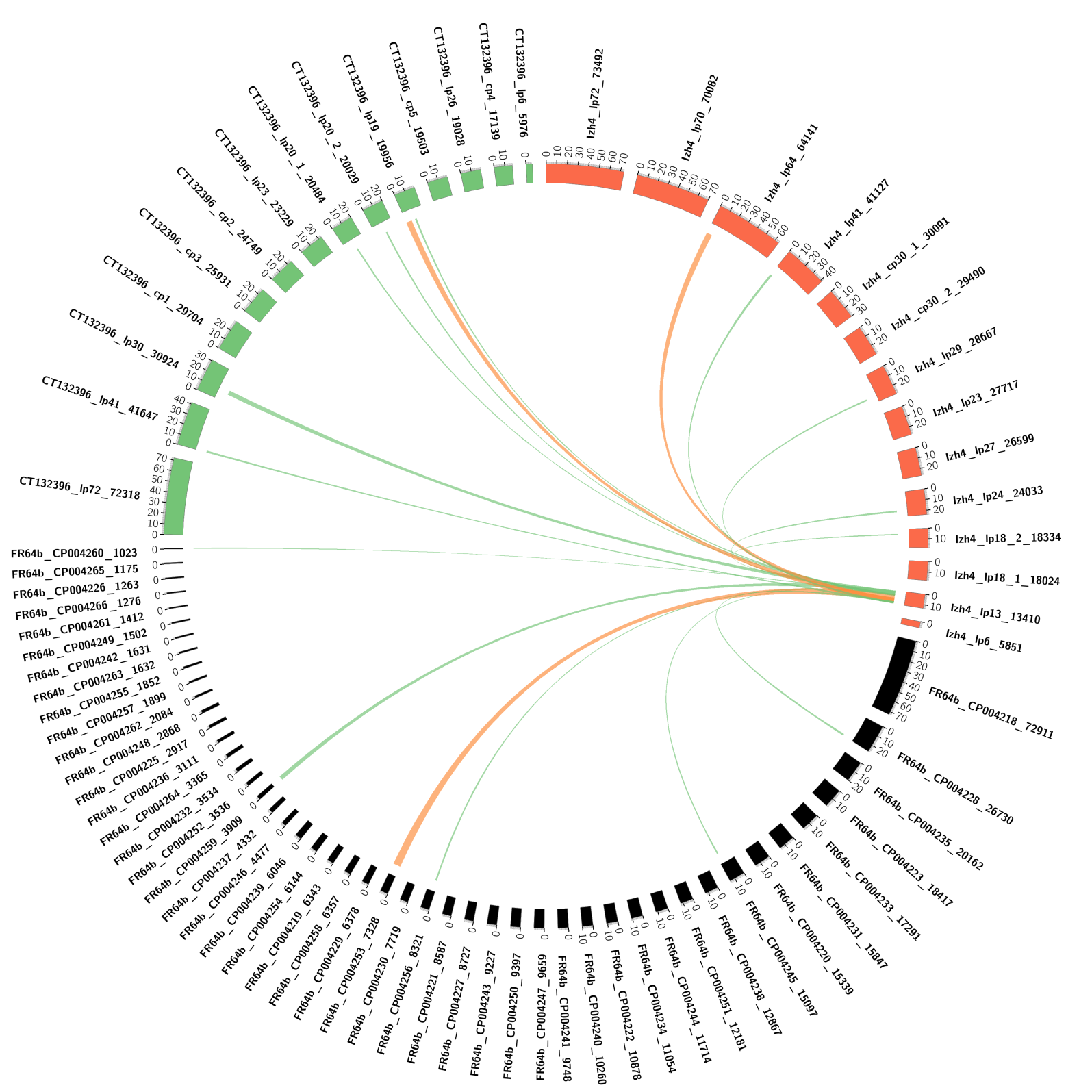


Supplemental Figure S29. Izh-4 lp6 is similar to analogous small plasmids in CT13-2396 and FR64b. This is a case where a small contig in FR64b represents real plasmid.


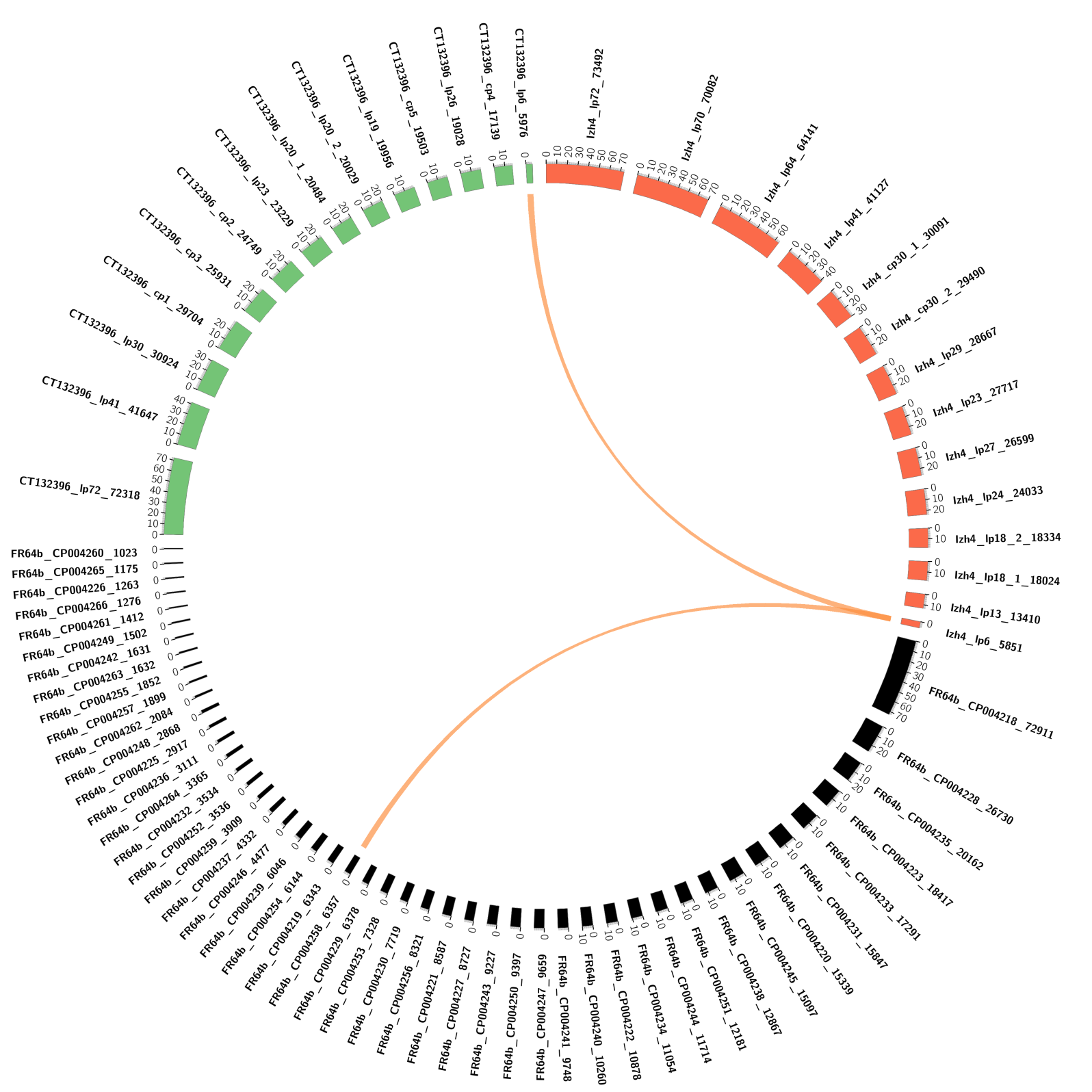


**Izh-4 lp6**
