# Supplementary material for: Whole genome sequencing of Borrelia miyamotoi isolate Izh-4: reference for a complex bacterial genome
Source: BMC Genomics. 2020 Jan 6;21:16. doi: 10.1186/s12864-019-6388-4 (PMC6945570; doi:10.1186/s12864-019-6388-4)
Supplement: Supplementary file 3 — Additional file 3: Figures S30 - S32 and Table S2. Nucleotide sequences of chromosomes of four B. miyamotoi genomes (3-USA, 1-Japan) were aligned to the chromosome of Izh-4 by Mummer and positions of regions containing structural variation were detected by NucDiff and visualized in the IGV browser. Figure S33. Similarity of regions which contain the PF57/62 genes located on lp18–1 and lp18–2 plasmids of isolate Izh-4. Figure S34. Similarity of regions which contain PF57/62 genes located on lp29 and lp27 plasmids of Izh-4 isolate. Figure S35. Alignment of the intergenic region located upstream of the expressed Vmp gene on lp41 of FR64b, Izh-4, CT13–2396, and LB-2001. Figure S36. Similarity of the right end of plasmids lp41 and lp23. [file 12864_2019_6388_MOESM3_ESM.docx]

**Supplementary materials**

Supplemental Figure 30. Nucleotide sequences of chromosomes of four *B. miyamotoi* genomes (3-USA, 1-Japan) were aligned to Izh-4 chromosome by Mummer and positions with regions containing repeats were detected by NucDiff and visualized in the IGV browser.


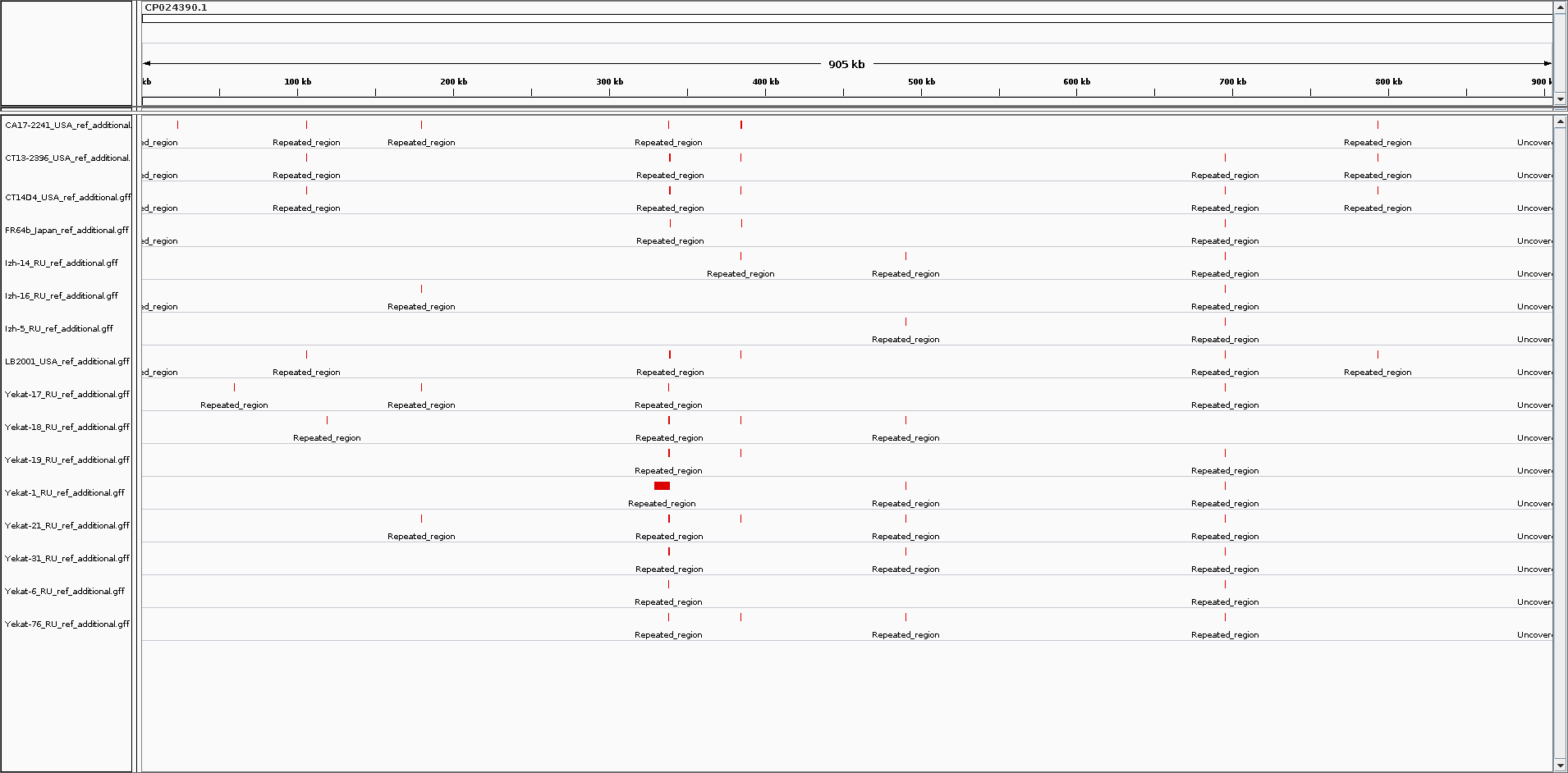


Supplemental Figure 31. Nucleotide sequences of chromosomes of four *B. miyamotoi* genomes (3-USA, 1-Japan) were aligned to Izh-4 chromosome by Mummer and positions with structural variation (duplications, deletions, insertions, tandem repeats variations) were detected by NucDiff and visualized in the IGV browser.


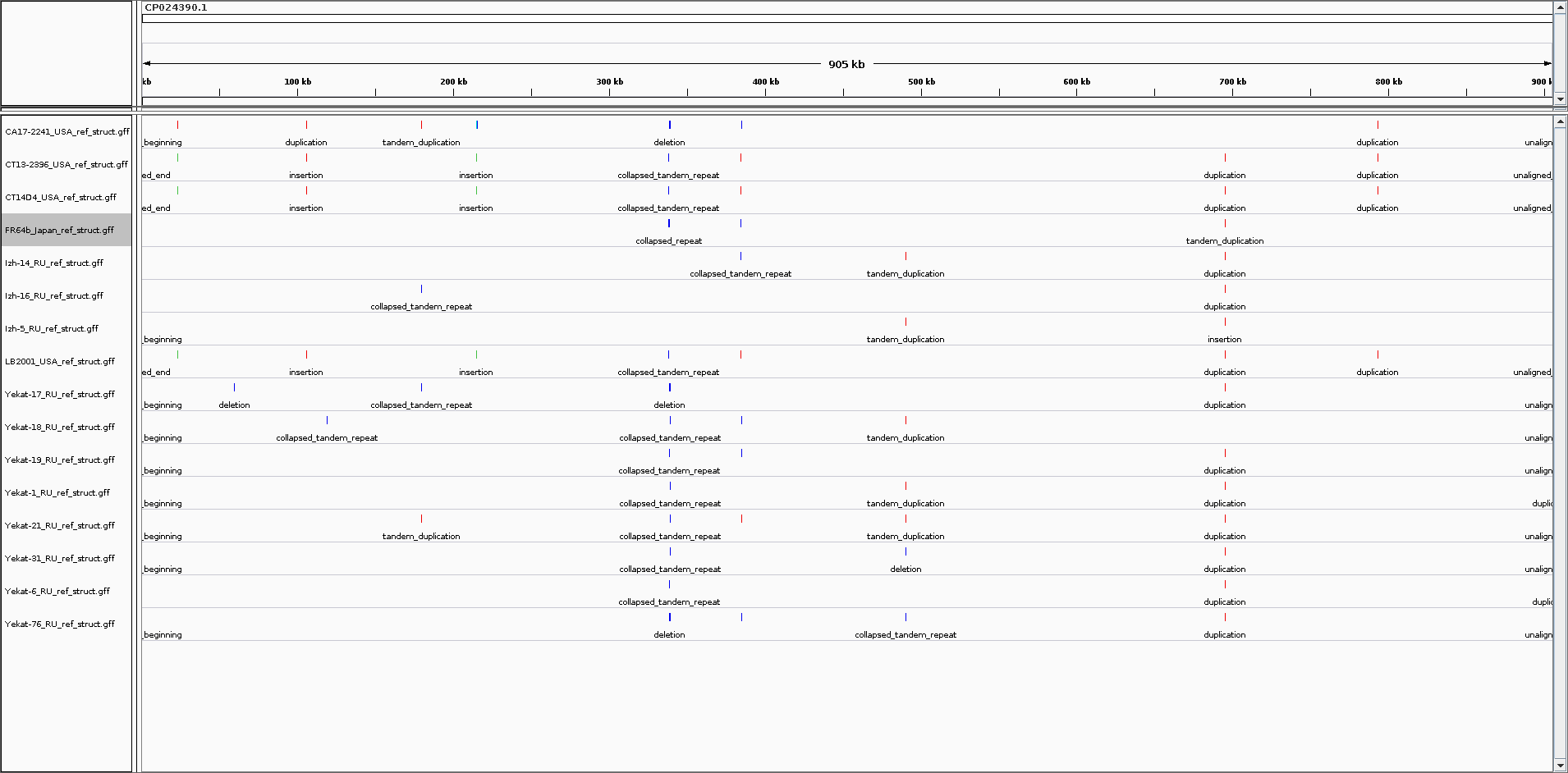


Supplemental Figure 32. Nucleotide sequences of chromosomes of four *B. miyamotoi* genomes (3-USA, 1-Japan) were aligned to Izh-4 chromosome by Mummer and positions with small and medium local differences (simple insertions (red), simple deletions (blue), simple substitution (green)) were detected by NucDiff and visualized in the IGV browser.


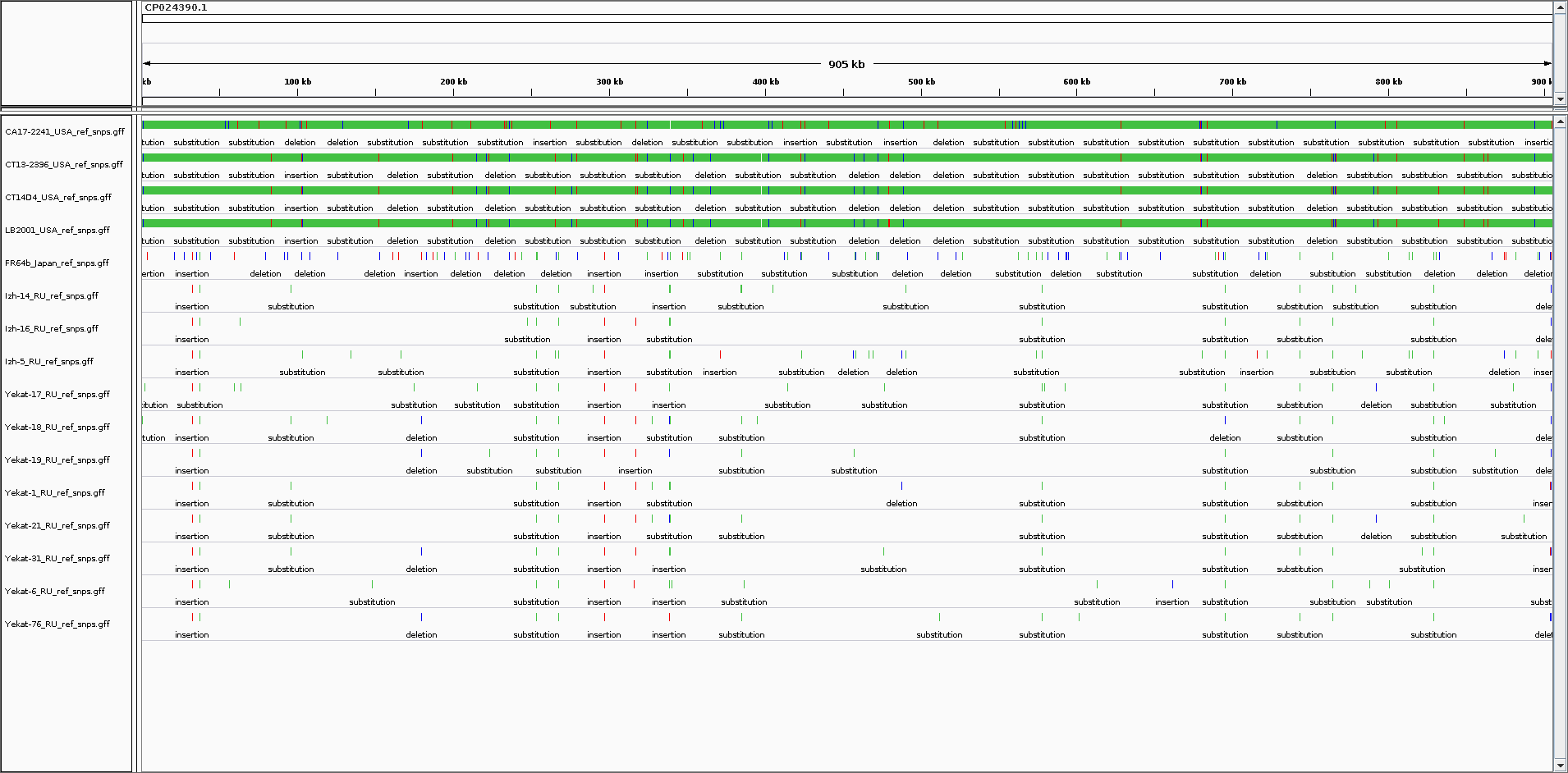


Supplemental Table 2. Nucleotide sequences of chromosomes of four *B. miyamotoi* genomes (3-USA, 1-Japan) were aligned to Izh-4 chromosome by Mummer and differences between Izh-4 and the query genome were detected by NucDiff.

| **MAIN INFORMATION:** | | | | | | | | | | | | | **DETAILED INFORMATION:** | | | | | | | | | | | | | | | | | | | | | |
| --- | --- | --- | --- | --- | --- | --- | --- | --- | --- | --- | --- | --- | --- | --- | --- | --- | --- | --- | --- | --- | --- | --- | --- | --- | --- | --- | --- | --- | --- | --- | --- | --- | --- | --- |
| Name of strain | Total number of differences | Insertions | Deletions | Substitutions | Translocations | Relocations | Reshufflings | Reshuffled blocks | Inversions | Unaligned sequences | Uncovered ref regions num | Uncovered ref regions len | substitution | gap | insertion | duplication | tandem_duplication | unaligned_beginning | unaligned_end | inserted_gap | deletion | collapsed_repeat | tandem_collapsed_repeat | translocation | translocation-insertion | translocation-insertion_ATGCN | translocation-inserted_gap | translocation-overlap | circular_genome_start | relocation | relocation-insertion | relocation-insertion_ATGCN | relocation-inserted_gap | relocation-overlap |
| CA17-2241 USA | 19042 | 399 | 339 | 18303 | 0 | 0 | 0 | 0 | 1 | 0 | 2 | 89 | 18303 | 0 | 393 | 3 | 1 | 1 | 1 | 0 | 337 | 1 | 1 | 0 | 0 | 0 | 0 | 0 | 0 | 0 | 0 | 0 | 0 | 0 |
| CT13-2396 USA | 18395 | 416 | 322 | 17657 | 0 | 0 | 0 | 0 | 0 | 0 | 2 | 39 | 17657 | 0 | 410 | 3 | 1 | 1 | 1 | 0 | 321 | 0 | 1 | 0 | 0 | 0 | 0 | 0 | 0 | 0 | 0 | 0 | 0 | 0 |
| CT14D4 USA | 18405 | 418 | 323 | 17664 | 0 | 0 | 0 | 0 | 0 | 0 | 2 | 39 | 17664 | 0 | 412 | 3 | 1 | 1 | 1 | 0 | 322 | 0 | 1 | 0 | 0 | 0 | 0 | 0 | 0 | 0 | 0 | 0 | 0 | 0 |
| LB2001 USA | 18409 | 418 | 323 | 17668 | 0 | 0 | 0 | 0 | 0 | 0 | 2 | 39 | 17668 | 0 | 412 | 3 | 1 | 1 | 1 | 0 | 322 | 0 | 1 | 0 | 0 | 0 | 0 | 0 | 0 | 0 | 0 | 0 | 0 | 0 |
| FR64b Japan | 122 | 17 | 51 | 54 | 0 | 0 | 0 | 0 | 0 | 0 | 2 | 403 | 54 | 0 | 16 | 0 | 1 | 0 | 0 | 0 | 49 | 1 | 1 | 0 | 0 | 0 | 0 | 0 | 0 | 0 | 0 | 0 | 0 | 0 |

Supplemental Figure 33. (A) Similarity of regions (1960bp with 99% identity) which contain the PF57/62 genes located on lp18-1 and lp18-2 plasmids of isolate Izh-4 based on BLAST analysis. * - pseudogene. (B) Schematic representation of pairwise alignment between ORFs CNO09_06450 (1576bp) located on plasmid lp18-1 and CNO09_06095 (1412bp) located on plasmid lp18-2. Despite a high similarity level between these ORFs (99%) several nucleotide variations and indels are present in the alignment (see Consensus Identity). The analysis was performed by Easyfig and Geneious v7.1.7.

**A**


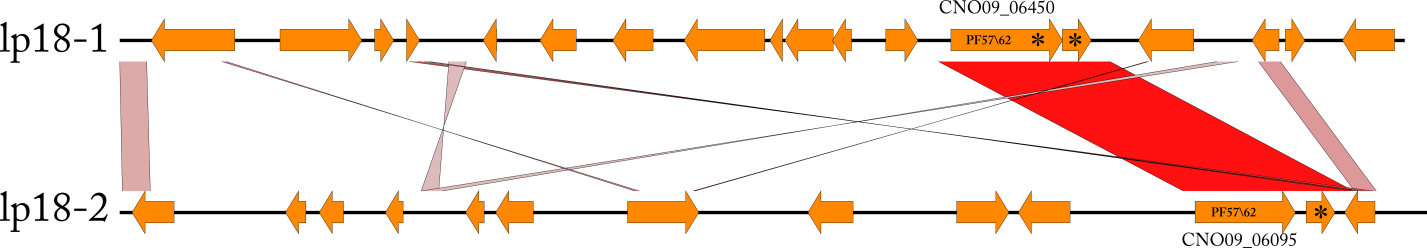


**B**


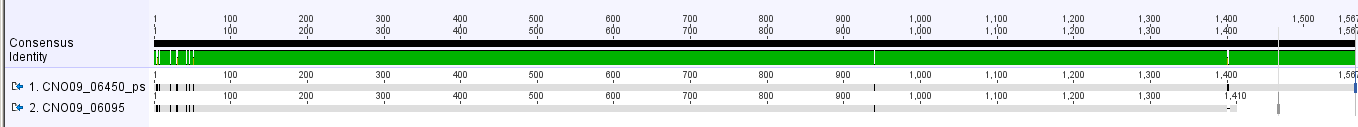


Supplemental Figure 34. (A) Similarity of regions (4800bp with 92% similarity) which contain PF57/62 genes located on lp29 and lp27 plasmids of isolate Izh-4 based on BLAST analysis. (B) Schematic representation of pairwise alignment between ORFs CNO09_05685 (1644bp) located on plasmid lp29 and CNO09_05835 (1644bp) located on plasmid lp27. Despite a high similarity level between these ORFs (99%) several nucleotide variations are present in the alignment (see Consensus Identity). The analysis was performed by Easyfig and Geneious v7.1.7.

**A**


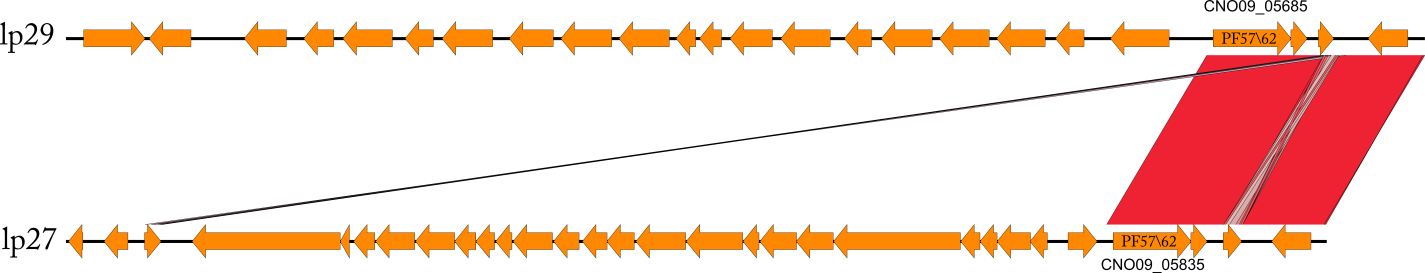


**B**


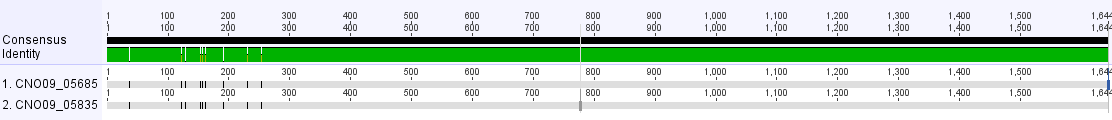


Supplemental Figure 35. Alignment of the intergenic region located upstream of the expressed Vmp gene on lp41 of FR64b, Izh-4, CT13-2396, and LB-2001. Areas of a Ribosome Binding Site (RBS) and the "-35" and "-10" sites are indicated by boxes.

RBS

-10

-35

Supplemental Figure 36. Similarity of the right end of plasmids lp41 and lp23. The red color indicates identical nucleotide sequences whilst the pink color shows similarity at a lower level (80 %) between different regions. VlpPS indicates pseudogene of Vlp.
