# Supplementary material for: Whole genome sequencing of Borrelia miyamotoi isolate Izh-4: reference for a complex bacterial genome
Source: BMC Genomics. 2020 Jan 6;21:16. doi: 10.1186/s12864-019-6388-4 (PMC6945570; doi:10.1186/s12864-019-6388-4)
Supplement: Supplementary file 4 — Additional file 4: Figure S37. PF32 phylogeny. Figure S38. PF49 phylogeny. Figure S39. PF50 phylogeny. Figure S40. PF57/62 phylogeny. [file 12864_2019_6388_MOESM4_ESM.docx]

**Supplementary materials**

Supplemental Figure 37. PF32 phylogeny. The positions of Izh-4 sequences are highlighted.

Supplemental Figure 38. PF49 phylogeny.

Supplemental Figure 39. PF50 phylogeny.


Supplemental Figure 40. PF57/62 phylogeny.
