# Supplementary material for: Whole genome sequencing of Borrelia miyamotoi isolate Izh-4: reference for a complex bacterial genome
Source: BMC Genomics. 2020 Jan 6;21:16. doi: 10.1186/s12864-019-6388-4 (PMC6945570; doi:10.1186/s12864-019-6388-4)

**Supplemental Figure 41**.

Schematic dot plots of PacBio ("PC") and ONT ("ONT") contigs with corresponding plasmid names aligned against itself using FlexiDot v1.06. The length of X and Y axes for each plot is equal. The green lines indicate reverse similarities while the black ones indicate the forward similarities. For example, lp72, lp29, lp27, lp24, lp18-1 are characterized by overhang duplicated regions at both 5' and 3' ends, while lp18-2, lp13, lp6 has overhang duplicated regions at either 5' or 3' ends. The detailed inspection, defining of the break points (for example, red dots on lp72) from one or two sides for each contig and the trimming of overlapped end-to-end regions was performed manually using Unipro UGENE v1.32.0 software [1].

For contigs corresponding to chromosome, lp64, lp41, lp23 there were no overhang duplicated regions at both 5' and 3' ends detected after *de novo* assembly, because it was based on ONT data only, as described in the Results section "*B. miyamotoi* strain, genome sequencing and assembly". However, manual inspection of mapped PacBio reads on both the left and right ends of these contigs shows the presence to telomere sequences. Contigs of cp30-1 and cp30-2 plasmids has overlaps indicating circular plasmids.

**References**

1. Okonechnikov K, Golosova O, Fursov M, team U: Unipro UGENE: a unified bioinformatics toolkit. Bioinformatics 2012, 28(8):1166-1167.


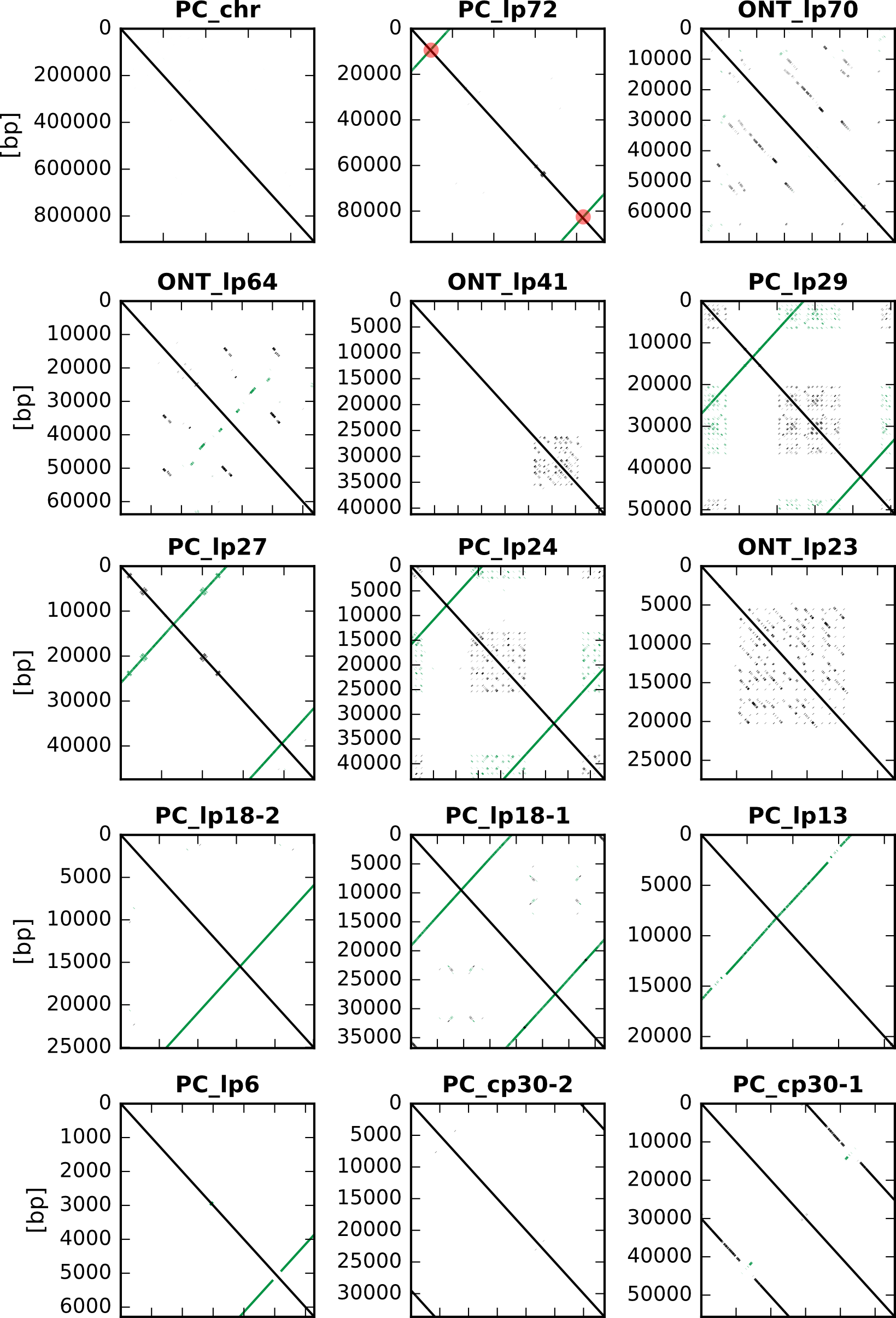

Supplement: Supplementary file 5 — Additional file 5: Figure S41. Schematic dot plots of PacBio and ONT contigs with corresponding plasmid names aligned against itself. [file 12864_2019_6388_MOESM5_ESM.docx]
